# Resident-invader dynamics of similar strategies in fluctuating environments

**DOI:** 10.1007/s00285-020-01532-8

**Published:** 2020-09-07

**Authors:** Yuhua Cai, Stefan A. H. Geritz

**Affiliations:** Department of Mathematics and Statistics, University of Helsinki, PO Box 68, 00014 Helsinki, Finland

**Keywords:** Adaptive dynamics, Invasion dynamics, Environmental feedback, Environmental stochasticity, Stochastic fast-slow systems, 34F05, 37N25, 92D15, 92D25

## Abstract

We study resident-invader dynamics in fluctuating environments when the invader and the resident have close but distinct strategies. First we focus on a class of continuous-time models of unstructured populations of multi-dimensional strategies, which incorporates environmental feedback and environmental stochasticity. Then we generalize our results to a class of structured population models. We classify the generic population dynamical outcomes of an invasion event when the resident population in a given environment is non-growing on the long-run and stochastically persistent. Our approach is based on the series expansion of a model with respect to the small strategy difference, and on the analysis of a stochastic fast-slow system induced by time-scale separation. Theoretical and numerical analyses show that the total size of the resident and invader population varies stochastically and dramatically in time, while the relative size of the invader population changes slowly and asymptotically in time. Thereby the classification is based on the asymptotic behavior of the relative population size, and which is shown to be fully determined by invasion criteria (i.e., without having to study the full generic dynamical system). Our results extend and generalize previous results for a stable resident equilibrium (particularly, Geritz in J Math Biol 50(1):67–82, 2005; Dercole and Geritz in J Theor Biol 394:231-254, 2016) to non-equilibrium resident population dynamics as well as resident dynamics with stochastic (or deterministic) drivers.

## Introduction

Two important issues in the framework of adaptive dynamics are which mutant strategies can invade a population of given resident strategies, and what would be the population dynamical outcomes of an invasion event. The long-term growth rate of a newly arrived and initially rare mutant with strategy *y* in the environment generated by a population of resident strategy *x* (i.e., invasion fitness of mutant *y* in resident *x*) determines whether an invasion event may occur or not (Metz et al. 1992, 1996; Dieckmann and Law 1996; Geritz et al. 1997, 1998). If *y* can invade *x* but not *vice versa*, does it mean that *y* will eventually take over *x* and becomes the new resident? In general, this need not happen as examples of unprotected coexistence and the “resident strikes back” phenomenon show (see e.g., Doebeli 1998; Parvinen 1999; Mylius and Diekmann 2001; Dercole et al. 2002). However, if *y* is close to, but not identical to *x*, Geritz (2005) and Dercole and Geritz (2016) have shown that invasion without back-invasion generically implies substitution, and mutual invasion generically leads to coexistence in a broad class of population models. They found that the generic population dynamical outcomes of an invasion event of similar strategies are determined by invasion criteria alone, i.e., without having to study the full generic dynamical system.

The focus of Geritz (2005) and Dercole and Geritz (2016) is on resident-invader dynamics of similar strategies in a constant environment, providing that the resident population in a large ecological community is at a hyperbolic attracting steady state. Recently, there has been considerable interest in questions related to evolution of phenotypic diversity in fluctuating environments (Kussell and Leibler 2005; Kisdi and Liu 2006; Geritz et al. 2007; Schreiber 2012b; Wakano and Iwasa 2013; Ripa and Dieckmann 2013; Melbinger and Vergassola 2015; Sæther and Engen 2015; Ferris and Best 2019). The purpose of this paper is to extend and generalize the results of Geritz (2005) and Dercole and Geritz (2016) to environmental fluctuations due to non-equilibrium population dynamics (e.g., cycle, quasiperiodic trajectory and chaos) or environmental stochasticity. This requires (i) considering an explicit model that takes account of environmental feedback and environmental stochasticity, and (ii) specifying the population dynamics of residents in fluctuating environments.

A population affects its environment, and the environment in turn affects the population. Such an environmental feedback loop characterizes the interaction of populations with their environments and plays a central role in their ecological and evolutionary dynamics (Metz et al. 1996, 2008; Meszéna and Metz 1999; Kisdi and Geritz 2016; Lion 2018b). Examples of feedback variables are: interacting species (e.g., predator, prey and food); different classes in a population who is structured by age, sex, spatial location, etc; and physical variables (e.g., temperature and humidity) which have been shown that they may provide a feedback between ecological communities and local physical patterns in some concrete examples (see the Introduction of Benaïm and Schreiber (2019) and references therein). These feedback variables need to be modelled with their own dynamics. Environmental stochasticity, however, generally refers to effects of fluctuations in external factors (which can be biotic or abiotic) on model parameters (Nisbet and Gurney 1982; Kliemann 1983; Chesson 1986; Tuljapurkar 1990). These external factors drive the dynamics of the aforementioned feedback variables but not *vice versa*. This is what differentiates environmental stochasticity from environmental feedback.

A population of a resident strategy in a fluctuating environment generally requires to be non-growing on the long-run and stochastically persistent. The non-growing property means that the long-term growth rate of the resident population in a given environment is zero. Stochastic persistence is an important concept to characterize population dynamics in fluctuating environments, which captures the property that if initially present, even if only in small size, then it will be present over arbitrary long periods of time in a stochastic sense. There are serval different (not necessarily equivalent) definitions of stochastic persistence (see e.g., a review in Schreiber 2012a). In this paper, we use the definition of Schreiber et al. (2011) and Benaïm (2018) which asserts that the probability of a population being near extinction is arbitrarily small.

The rest of this paper is organized as follows. In Sect. 2, we focus on the analysis of resident-invader dynamics of a class of unstructured population models. In Sect. 2.1, a class of polymorphic unstructured population models for multi-dimensional strategies and an explicit formulation of environmental feedback and environmental stochasticity is presented. In Sect. 2.2, we review some basic concepts used in this paper. In Sect. 2.3, we specify when a population of a given strategy is a resident. Section 2.4 gives the definition of invasion fitness. In Sect. 2.5, we present the basic analysis of invasion dynamics of similar strategies. Particularly, in Sects. 2.5.2 and 2.5.3, we classify the generic population dynamical outcomes of an invasion event. In Sect. 3, we illustrate how to generalize the results of the unstructured population models to a class of structured population models. In Sect. 4, we apply our results to examples of evolving bacteria in a chemostat, Lotka-Volterra competition, structured SIRS epidemic dynamics, and the evolution of timidity of the prey in a prey-predator model. The first two examples are designed to highlight different ways that our results can be used. The third example is designed to illustrate how a concrete structured population model can be reformulated into our framework. The last example is designed to demonstrate that our results are applicable to the evolution with the non-equilibrium resident dynamics. In Sect. 5, we conclude with discussing how our results relate to the existing literature and what possible generalizations are. Proofs are given in the “Appendix”.

Before proceeding with the analysis, we introduce some default notations used in this paper. Let ${\mathcal {X}}$ be the strategy space, ${\mathcal {N}}$ be the space of non-negative population sizes, ${\mathcal {E}}$ be the space of feedback variables and $\varTheta$ be the space of external factors. Each of these spaces is assumed to be a subset of a normed vector space. $f^{(i)}$ is the $i{\mathrm {th}}$ derivative of function *f* with respect to its first argument, and $f^{(i,j)}$ is the $i{\mathrm {th}}$ derivative of function *f* with respect to its first argument and the $j{\mathrm {th}}$ derivative with respect to its second argument. The superscript $\top$ means transpose of a vector or a matrix.

## Unstructured population models

### The polymorphic population model

Consider a population of strategies $x_{1},\dots ,x_{k}\in {\mathcal {X}}$ whose sizes at time *t* are given by $n_{1,t},\dots ,n_{k,t}\in {\mathcal {N}}$, respectively. The population of $x_{1},\dots ,x_{k}$ interact with $e_{t}\in {\mathcal {E}}$ that describes the feedback environment. Generally, $e_{t}$ may correspond to interacting species, different classes of the population, physical factors, or any combination thereof. $n_{1,t},\dots ,n_{k,t}$ as well as $e_{t}$ may be influenced by a stochastic driver $\theta _{t}\in \varTheta$ that describes external factors. The per-capita growth rate *f* of individuals with strategy $x_{i}$ depends on the current environmental condition $(e_{t},\theta _{t})$. Under these considerations, the dynamics of the polymorphic unstructured population are given by

2.1a $$\begin{aligned} {\dot{n}}_{i,t}=f(x_{i},e_{t},\theta _{t})n_{i,t},\ \ i=1,\dots ,k \end{aligned}$$

where the dot denotes differentiation with respect to time variable *t*. Notice that for a given environmental condition $(e_{t},\theta _{t})$, (2.1a) is linear in the population size. All the nonlinearity of (2.1a) comes from how the environment $e_{t}$ depends on the population sizes.

Before introducing the formulation of the dynamics of $e_{t}$, let us look at how the environment $e_{t}$ depends on the population sizes in the following two deterministic models.

#### Example 1

Lehtinen and Geritz (2019) studied the evolution of timidity in a prey species whose predator has cannibalistic tendencies, in which the population dynamics is given by

$$\begin{aligned} \begin{array}{ll} (\text {prey } i) &{}{\dot{n}}_{i,t} =n^{F}_{i,t}g\bigg (\sum \limits _{j}n^{F}_{j,t}\bigg ) -\mu n_{i,t}-\beta n^{F}_{i,t}p^{S}_{t}, \\ (\text {adult predator})&{}{\dot{p}}_{t} =\frac{1}{T}J_{t}-\delta p_{t}, \end{array} \end{aligned}$$

where the reproduction of prey *i* is limited by competition (of resources, territories, breeding sites, etc.) among the foraging prey $n^{F}_{i,t}=\frac{n_{i,t}}{1+x_{i}p_{t}}$ with the per-capita birth rate $g\big (\sum _{j}n^{F}_{j,t}\big )=a-c\sum _{j}n^{F}_{j,t}$, a searching adult predator $p^{S}_{t}=\frac{p_{t}}{1+\beta h\sum _{j}n^{F}_{j,t}}$ captures the foraging prey at the rate $\beta$, the maturation of juvenile predators $J_{t}=\frac{\gamma \beta p^{S}_{t}\sum _{j}n^{F}_{j,t}}{\sigma +(1-\lambda )\alpha p^{S}_{t}}$ with the mean time *T* depends on the cannibalistic pressure they experience throughout their juvenile period, and the per-capita death rate of the adult predator and the predation-independent per-capita death rate of the prey are $\delta$ and $\mu$, respectively. The prey strategies differ only in their level of timidity $x_{i}\in {\mathbb {R}}_{+}:={\mathcal {X}}$. Other parameters are all positive constant. Let

$$\begin{aligned} \begin{array}{ll} {(\text {adult predators})}&{}e_{1,t} = p_{t}, \\ {(\text {foraging preys})}&{}e_{2,t} = \sum _{j}n^{F}_{j,t}=\sum _{j}\frac{1}{1+x_{j}e_{1,t}}n_{j,t}, \end{array} \end{aligned}$$

then

$$\begin{aligned} {\dot{e}}_{1,t}=\sum _{j}\frac{\gamma \big (1+\beta he_{2,t}\big )\frac{\beta e_{1,t}}{(1+\beta he_{2,t})(1+x_{j}e_{1,t})}}{T\big (\sigma (1+\beta h e_{2,t})+(1-\lambda )\alpha e_{1,t}\big )}n_{i,t}-\delta e_{1,t}. \end{aligned}$$

Since $e_{1,t}$ is an implicit function of the prey sizes and $e_{2,t}$ is an explicit function of $e_{1,t}$, the environmental variable $e_{2,t}$ becomes nonlinear in the prey sizes. Thus, the per-capita population growth rate *f* of prey type *i* in the nonlinear environment $(e_{1,t},e_{2,t}):=e_{t}$ is given by

$$\begin{aligned} f(x_{i},e_{t})=\frac{g(e_{2,t})}{1+x_{i}e_{1,t}}-\mu -\frac{\beta e_{1,t}}{(1+x_{i}e_{1,t})(1+\beta he_{2,t})}. \end{aligned}$$

#### Example 2

Dercole and Rinaldi (2002) and Dercole (2003) studied the evolution of cannibalistic strategies, in which the interactions between *k* cannibalistic consumer sub-populations with sizes $n_{i,t}$ and strategies $x_{i}\in {\mathbb {R}}_{+}:={\mathcal {X}}$, $i=1,\dots ,k$, are described by

$$\begin{aligned} \begin{array}{ll} \quad &{}{\dot{n}}_{i,t}=n_{i,t}\bigg (\gamma \frac{a_{0}(x_{i})n_{0}+\sum _{j}a(x_{i},x_{j})n_{j,t}}{1+h_{0}(x_{i})a_{0}(x_{i})n_{0}+\sum _{j}h(x_{i})a(x_{i},x_{j})n_{j,t}} \\ {(\text {type } i)}&{}\qquad \qquad -\sum \limits _{j}\frac{a(x_{j},x_{i})n_{j,t}}{1+h_{0}(x_{j})a_{0}(x_{j})n_{0}+\sum _{\ell }h(x_{j})a(x_{j},x_{\ell })n_{\ell ,t}} \\ &{}\qquad \qquad -c\sum \limits _{j} n_{j,t}\bigg ), \end{array} \end{aligned}$$

where the conversion factor $\gamma$, the competition coefficient *c*, and the size of a common resource $n_{0}$ are assumed to be positive constant, while the attack rate *a* (and $a_{0}$) and the handling time *h* (and $h_{0}$) depend upon the strategies. In the bracket of the equation, the first term is reproduction through the harvesting of the common resource and cannibalism toward the sub-population *j*, the second term is mortality due to cannibalism, and the last term is mortality due to competition. For a given strategy $x_{i}$, let

$$\begin{aligned} \begin{array}{ll} \text {(harvested food)}&{}\quad e_{21,t}(x_{i}) = \sum \limits _{j}a(x_{i},x_{j})n_{j,t}, \\ \text {(handling times)}&{}\quad e_{22,t}(x_{i}) = \sum \limits _{j}h(x_{i})a(x_{i},x_{j})n_{j,t}, \\ \text {(cannibalism)}&{}\quad e_{23,t}(x_{i}) = \sum \limits _{j}\frac{a(x_{j},x_{i})}{1+h_{0}(x_{j})a_{0}(x_{j})n_{0}+e_{22,t}(x_{j})}n_{j,t}, \\ \text {(competition)}&{}\quad \qquad e_{24,t} = \sum \limits _{j}c n_{j,t}. \end{array} \end{aligned}$$

Here the environmental variables $e_{21,t}$, $e_{22,t}$ and $e_{24,t}$ are all linear in the population sizes, but the environmental variable $e_{23,t}$ is a nonlinear and explicit function of $e_{22,t}$ which leads to the nonlinearity of $e_{23,t}$ in the population sizes. Thus, the per-capita population growth rate *f* of sub-population *i* with a given strategy $x_{i}$ in the nonlinear environment $(e_{21,t},e_{22,t},e_{23,t},e_{24,t}):=e_{t}$ is given by

$$\begin{aligned} f(x_{i},e_{t})=\gamma \frac{a_{0}(x_{i})n_{0}+e_{21,t}(x_{i})}{1+h_{0}(x_{i})a_{0}(x_{i})+e_{22,t}(x_{i})}-e_{23,t}(x_{i})-e_{24,t} \end{aligned}$$

(N.B., $e_{21,t}$, $e_{22,t}$ and $e_{23,t}$ are functions of strategy $x_{i}$ and the strategy of sub-population type *i* has infinitely many choices, which causes an infinite-dimensional environment $e_{t}$ to appear. We will provide more interpretations for the case of infinite-dimensional environment below).

Following Diekmann et al. (2001) and Geritz (2005), the environment $e_{t}$ is assumed to be a linear function of the population sizes

$$\begin{aligned} e_{t}=\sum _{j}I(x_{j})n_{j,t}, \end{aligned}$$

where $I(x_{j})$ describes the environmental impact of a single individual with strategy $x_{j}$. However, this assumption excludes many interesting models with nonlinear environments such as the previous two examples. In Example 1, the predator size necessarily is an environmental variable for the preys, and the fraction of time individual prey spend foraging depends on the adult predators. In Example 2, the harvested food, the total handling times, and the compete-caused and cannibalize-caused death are environmental variables for the sub-population *i*, in which the cannibalize-caused death depends on the total handling times. We note that different environmental variables may have different forms even in the same model. For instance, in Example 1, $e_{1,t}$ is govern by a differential equation but $e_{2,t}$ is a function of $e_{1,t}$. For these reasons, we describe the environment by implicit (differential) equations.

**Fig. 1:**
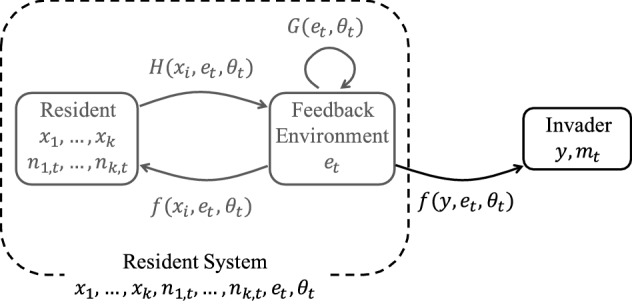
Diagram of the interaction between the resident population and the feedback environment and the one-directional impact on the invader population

In this paper, we consider an explicit formulation of the dynamics of the feedback environment $e_{t}=(e_{1,t},e_{2,t})$ as illustrated in Fig. 1, which is given by

2.1b $$\begin{aligned} \begin{aligned} {\dot{e}}_{1,t}&= G_{1}(e_{t},\theta _{t})+\sum _{j}H_{1}(x_{j},e_{t},\theta _{t})n_{j,t},\\ e_{2,t}&= G_{2}(e_{t},\theta _{t})+\sum _{j}H_{2}(x_{j},e_{t},\theta _{t})n_{j,t}. \end{aligned} \end{aligned}$$

The dynamics of $e_{1,t}$ is given by a differential equation, while $e_{2,t}$ is an implicit function of the current state of the system. The functions $G_{1}$ and $G_{2}$ describe the intrinsic dynamics of the virgin environment (the environment unaffected by the population of $x_{1},\dots ,x_{k}$). Given the current environment, the functions $H_{1}$ and $H_{2}$ describe the environmental impact of a single individual with a given strategy. Consequently, the second terms of (2.1b) gives the total environmental impact of the entire population of $x_{1},\dots ,x_{k}$. These functions all involve the current environmental condition $(e_{t},\theta _{t})$. Here we would like to highlight that $e_{2,t}$ is assumed to be unique defined by the implicit equation. As Example 2 and “Appendix A” show, $H_{2}$ can be a function of $e_{2,t}$, but $e_{2,t}$ is still unique defined. Alternatively, by allowing $e_{t}$ to be infinite-dimensional, we can also incorporate it into (2.1b) (Geritz 2005). For instance, in Example 2 and the model in Sect. 4.2, where the feedback variables at any time are a function of strategy and where $f(x_{i},e_{t},\theta _{t})$ involves the evaluation of $e_{t}$ in $x_{i}$ (ref to “Appendix A” and Sect. 4.2, respectively).

To consider external factors, we assume that $\{\theta _{t}\}_{t\ge 0}$ is a continuous time Markov process which is defining on an underlying probability space and taking value in $\varTheta$. From the definition of Markov process, the future state is independent of the past and depends only the present, which is a natural consideration in reality and contains a large number of ecologically interpretable factors. There are many ways to construct a Markov process, e.g., a pure jump process and a process generated by stochastic differential equations. In this paper, we assume that the process $\{\theta _{t}\}_{t\ge 0}$ is governed by the following stochastic differential equation:

2.1c $$\begin{aligned} {\dot{\theta }}_{t}=A(\theta _{t})+B(\theta _{t}){\dot{W}}_{t}, \end{aligned}$$

where ${\dot{W}}_{t}$ is a white noise, namely $W_{t}$ is a Brownian motion defined on a complete probability space $(\varOmega ,{\mathscr {F}},{\mathbb {P}})$ with a filtration $\{{\mathscr {F}}_{t}\}_{t\ge 0}$ satisfying the usual conditions (i.e., it is right continuous and increasing while ${\mathscr {F}}_{0}$ contains all ${\mathbb {P}}$-null sets). (2.1c) can be calculated using one of two methods: Itô or Stratonovich, but in fact our results do not depend on the calculation methods used.

Model (2.1) is an extension of the deterministic population models used in Geritz (2005), Dercole and Rinaldi (2008) and Dercole and Geritz (2016), which is intuitive and interpretable in biology and covers a large class of ecological models with an explicit formulation of environmental feedback and environmental stochasticity. Meanwhile, model (2.1) can be viewed as a revision of the stochastic population model used in Kliemann (1983), but now takes account of strategies and feedback variables. From the application point of view, any complicated considerations of biological mechanisms can be formulated into functions *f*, $G_{1}$, $H_{1}$, $G_{2}$, $H_{2}$, *A* and *B*, and a certain level of smoothness is assumed to make them mathematically meaningful.

Regarding (2.1), we make the following assumptions:

**A1**: The Markov process $\{\theta _{t}\}_{t\ge 0}$ is ergodic with invariant probability measure $\nu$ on $\varTheta$.
**A2**: The partial derivatives $f^{(i,j)}$, $G^{(i)}_{1}$, $H^{(i,j)}_{1}$, $G^{(i)}_{2}$ and $H^{(i,j)}_{2}$ exit and are locally Lipschitz continuous and measurable in $(e,\theta )$ for all strategies and for all nonnegative integers $i,j\le 2$. And functions *A* and *B* are locally Lipschitz continuous and measurable in $\theta$.

Assumption **A1** indicates the process $\{\theta _{t}\}_{t\ge 0}$ is completely realized before we start to observe the dynamics of $(n_{1,t},\dots ,n_{k,t},e_{t})$. Both meanings of ergodicity and invariance are given in Sect. 2.2. Process $\{n_{1,t},\dots ,n_{k,t},e_{t}\}_{t\ge 0}$ is usually not Markovian. By Assumption **A1**, however, process $\{n_{1,t},\dots ,n_{k,t},e_{t},\theta _{t}\}_{t\ge 0}$ is Markovian under weak assumptions (ref to Arnold and Kliemann (1983, Lemma 2.1)). Thereby it allows us to utilize the excellent works in the theory of Markov processes for the analysis of the process $\{n_{1,t},\dots ,n_{k,t},e_{t},\theta _{t}\}_{t\ge 0}$. Assumption **A2** is a technical requirement to guarantee the existence of chain rule and justify the series expansion.

### Some basic concepts

In order to state our results, we review some basic concepts in Markov process and ergodic theory.

Throughout Sect. 2, let

$$\begin{aligned} Z_{t}=(n_{1,t},\dots ,n_{k,t},e_{t},\theta _{t}), \end{aligned}$$

which lies in space

$$\begin{aligned} {\mathcal {Z}}={\mathcal {N}}^{k}\times {\mathcal {E}}\times \varTheta . \end{aligned}$$

For any Borel set $C\subset {\mathcal {Z}}$, let

$$\begin{aligned} {\mathbb {P}}_{z}\{Z_{t}\in C\}={\mathbb {P}}\{Z_{t}\in C|Z_{0}=z\} \end{aligned}$$

be the probability of $Z_{t}$ in *C* given $Z_{0}=z\in {\mathcal {Z}}$. Let $\{{\mathcal {T}}_{t}\}_{t\ge 0}$ be the associated semigroup of Markov process $\{Z_{t}\}_{t\ge 0}$ on ${\mathcal {Z}}$ defined by conditional expectations:

$$\begin{aligned} {\mathcal {T}}_{t}h(z)={\mathbb {E}}_{z}\big [h(Z_{t})\big ] ={\mathbb {E}}\big [h(Z_{t})|Z_{0}=z\big ], \end{aligned}$$

for bounded and measurable function $h: {\mathcal {Z}}\mapsto {\mathbb {R}}$. Assumption **A2** implies that the Markov semigroup $\{{\mathcal {T}}_{t}\}_{t\ge 0}$ is ${\mathscr {C}}_{b}({\mathcal {Z}})$-*Feller* (Benaïm 2018), i.e., operator ${\mathcal {T}}_{t}$ takes bounded continuous functions *h* on ${\mathcal {Z}}$ to bounded continuous functions ${\mathcal {T}}_{t}h$ on ${\mathcal {Z}}$ for all $t\ge 0$. A probability measure $\mu$ on ${\mathcal {Z}}$ is *invariant* for $\{Z_{t}\}_{t\ge 0}$ (or $\{{\mathcal {T}}_{t}\}_{t\ge 0}$) if

$$\begin{aligned} \int _{{\mathcal {Z}}}{\mathcal {T}}_{t}h(z)\mu (dz)=\int _{{\mathcal {Z}}}h(z)\mu (dz), \end{aligned}$$

for all bounded and measurable functions $h:{\mathcal {Z}}\mapsto {\mathbb {R}}$ and all $t\ge 0$. An invariant probability measure $\mu$ is *ergodic* if it can not be written as a non-trivial convex combination of other distinct invariant probability measures.

If $Z_{t}$ initially follows the distribution of an ergodic probability measure $\mu$, then Birkhoff’s ergodic theorem tells us that, in simple terms, the time average of a function of the process along the trajectories exists with probability one and equals the space average. More precisely, for all measurable functions $h:{\mathcal {Z}}\mapsto {\mathbb {R}}$ with $\int _{{\mathcal {Z}}}|h(z)|\mu (dz)<+\infty$,

$$\begin{aligned} \lim _{t\rightarrow +\infty }\frac{1}{t}\int _{0}^{t}h(Z_{s})ds =\int _{{\mathcal {Z}}}h(z)\mu (dz) \end{aligned}$$

with probability one.

### Resident and resident system

From (2.1a) with initial value $Z_{0}=z\in {\mathcal {Z}}$, a straightforward calculation shows that the species $x_{i}$ tends to increase in its population size if

$$\begin{aligned} \lim _{t\rightarrow +\infty }\frac{1}{t}\int _{0}^{t}f(x_{i},e_{s},\theta _{s})ds>0, \end{aligned}$$

and tends to decrease in its population size if

$$\begin{aligned} \lim _{t\rightarrow +\infty }\frac{1}{t}\int _{0}^{t}f(x_{i},e_{s},\theta _{s})ds<0, \end{aligned}$$

provided that the limit exists. Let $\mu$ be an invariant probability measure for process $\{Z_{t}\}_{t\ge 0}$. From Birkhoff’s ergodic theorem,

$$\begin{aligned} \lim _{t\rightarrow +\infty }\frac{1}{t}\int _{0}^{t}f(x_{i},e_{s},\theta _{s})ds =\lambda _{x_{i}}(\mu ) \end{aligned}$$

with probability one and for $\mu$-almost every *z*, where $\lambda _{x_{i}}(\mu )$ is the *per-capita growth rate of species* $x_{i}$ *with respect to* $\mu$:

$$\begin{aligned} \lambda _{x_{i}}(\mu )=\int _{{\mathcal {Z}}}f(x_{i},e,\theta )\mu (de,d\theta ). \end{aligned}$$

We now introduce two notations for further analysis. We define the *extinction set* of a population of strategies $x_{1},\dots ,x_{k}$

$$\begin{aligned} {\mathcal {Z}}_{0}=\big \{z\in {\mathcal {Z}}:\min _{i}n_{i}=0\big \} \end{aligned}$$

is the set which at least one species is absent, and the $\eta$-*neighborhood of the extinction set*

$$\begin{aligned} {\mathcal {Z}}_{\eta }=\big \{z\in {\mathcal {Z}}: \min _{i}n_{i}\le \eta \big \} \end{aligned}$$

is the set which at least one species has a size less than $\eta$.

Next, we specify when a population of strategies $x_{1},\dots ,x_{k}$ is a resident.

#### Definition 1

A population of strategies $x_{1},\dots ,x_{k}$ with corresponding size $\{n_{1,t},\dots ,n_{k,t}\}_{t\ge 0}$ in the environment $\{e_{t},\theta _{t}\}_{t\ge 0}$ is called a *resident* with respect to $\mu$ if $\mu$ is an invariant probability measure for process $\{Z_{t}\}_{t\ge 0}$ satisfying $\mu ({\mathcal {Z}}\setminus {\mathcal {Z}}_{0})=1$. Meanwhile,

(i) (*non-growing*) $\lambda _{x_{i}}(\mu )=0$ for every $i\in \{1,\dots ,k\}$, and
(ii) (*stochastically persistent*) for all $\varepsilon >0$, there exists a $\eta >0$ such that $\mu ({\mathcal {Z}}_{\eta })\le \varepsilon$.

#### Definition 2

The multi-tuple $(x_{1},\dots ,x_{k},n_{1,t},\dots ,n_{k,t},e_{t},\theta _{t})$ is called a *resident system* with respect to $\mu$ if $\mu$ is an invariant probability measure for process $\{Z_{t}\}_{t\ge 0}$ with properties satisfying Definition 1.

In Definition 1, the supports of required invariant probability measures should exclude the extinction set ${\mathcal {Z}}_{0}$. The first reason is that it is the technical requirement to guarantee the properties of *non-growing* and *stochastically persistence*. The second reason is from a probabilistic perspective that if a population initially follows an invariant probability measure $\mu$ with $\mu ({\mathcal {Z}}_{0})\ne 0$, then the extinction of the population is a non-zero probabilistic event on the long-term. From Definition 1, a resident is non-growing on the long-run and stochastically persistent, where the concept of persistence asserts that the probability of population size being near the extinction state is very small. In this paper, a resident (resp. a resident system) is characterized by an invariant probability measure defined on the state space. Actually, there might be more than one invariant probability measures, but we focus our attention on one of them. Each adequate invariant probability measure corresponds to a resident (resp. a resident system).

### Invasion fitness

Assume that $(x_{1},\dots ,x_{k},n_{1,t},\dots ,n_{k,t},e_{t},\theta _{t})$ is a resident system with an invariant probability measure $\mu$ satisfying Definition 1, which further determines the ecological environment for a mutant type $y\in {\mathcal {X}}$ with initially infinitesimal size $m_{t}\in {\mathcal {N}}$. Thus, the population dynamics of a newly arrived and initially rare mutant *y* is described by the following linear differential equation

2.2 $$\begin{aligned} {\dot{m}}_{t}=f(y, e_{t},\theta _{t})m_{t}. \end{aligned}$$

Notice that (2.2) holds as long as the mutant population remains rare. The tuple $(y,m_{t})$ is referred to an *invader*. Figure 1 shows the relationship of the invader $(y,m_{t})$ and the resident system $(x_{1},\dots ,x_{k},n_{1,t},\dots ,n_{k,t},e_{t},\theta _{t})$ where arrows with special indexes illustrate different impacts or contributions between them.

Formally, the *invasion fitness of mutant* *y* *in resident* $(x_{1},\dots ,x_{k})$ is given by

2.3 $$\begin{aligned} {\mathcal {S}}_{x_{1},\dots ,x_{k}}(y):=\lim _{t\rightarrow +\infty }\frac{\log m_{t}}{t}=\lim _{t\rightarrow +\infty }\frac{1}{t}\int _{0}^{t}f(y,e_{s},\theta _{s})ds=\lambda _{y}(\mu ) \end{aligned}$$

with probability one and for $\mu$-almost every *z* and for every positive size $m_{0}$ (Metz et al. 1992; Ferriere and Gatto 1995). The invader *y* dies out if ${\mathcal {S}}_{x_{1},\dots ,x_{k}}(y)<0$ and can spread if ${\mathcal {S}}_{x_{1},\dots ,x_{k}}(y)>0$. From Definition 1, if $y=x_{i}$ for any $i\in \{1,\dots ,k\}$, then we have

2.4 $$\begin{aligned} {\mathcal {S}}_{x_{1},\dots ,x_{k}}(x_{i})=\lambda _{x_{i}}(\mu )=0 \end{aligned}$$

which is so-called *the principle of selective neutrality of residents* in the framework of adaptive dynamics.

### Population dynamics of similar strategies

Consider now a population of two similar strategies (i.e., two close but distinct strategies) $x_{1}$, $x_{2}\in {\mathcal {X}}$ with corresponding sizes $n_{1,t}$, $n_{2,t}\in {\mathcal {N}}$ at time *t*. Without loss of generality, let strategies

$$\begin{aligned} x_{1}=x+\epsilon \xi _{1},\ x_{2}=x+\epsilon \xi _{2}, \end{aligned}$$

where $\xi _{1}\ne \xi _{2}$ with $\Vert \xi _{2}-\xi _{1}\Vert <+\infty$ and where small $\epsilon >0$ (N.B., here $\epsilon$ is different from the $\varepsilon$ used in Definition 1) and where *x* is a reference strategy. Biologically, $\epsilon$ can be viewed as the intensity of mutation, and $\xi _{i}$ is the direction of mutation with respect to the reference strategy *x*. Model (2.1) applied to the case of $k=2$ we further have a dimorphic population model. Obviously, $n_{1,t}$, $n_{2,t}$ and $e_{t}$ are functions of $x_{1}$ and/or $x_{2}$. Thus they are functions of $\epsilon$, which are denoted by $n^{\epsilon }_{1,t}$, $n^{\epsilon }_{2,t}$ and $e^{\epsilon }_{t}$, respectively.

To study the dimorphic population dynamics of $x_{1}$ and $x_{2}$ in fluctuating environments, following Meszéna et al. (2005) and Dercole and Geritz (2016), we introduce the total population size $N^{\epsilon }_{t}$ of $x_{1}$ and $x_{2}$ at time *t* and the relative population size $P^{\epsilon }_{t}$ of $x_{2}$ at time *t*, i.e.,

$$\begin{aligned} N^{\epsilon }_{t}=n^{\epsilon }_{1,t}+n^{\epsilon }_{2,t},\ P^{\epsilon }_{t}=\frac{n^{\epsilon }_{2,t}}{N^{\epsilon }_{t}}. \end{aligned}$$

Under these assumptions, we arrive at the following equations:

2.5a $$\begin{aligned} {\dot{N}}^{\epsilon }_{t}&= \big (f(x+\epsilon \xi _{1},e^{\epsilon }_{t},\theta _{t})(1-P^{\epsilon }_{t})+f(x+\epsilon \xi _{2},e^{\epsilon }_{t},\theta _{t})P^{\epsilon }_{t}\big )N^{\epsilon }_{t}, \end{aligned}$$

2.5b $$\begin{aligned} {\dot{P}}^{\epsilon }_{t}&= P^{\epsilon }_{t}(1-P^{\epsilon }_{t})\big (f(x+\epsilon \xi _{2},e^{\epsilon }_{t},\theta _{t})-f(x+\epsilon \xi _{1}, e^{\epsilon }_{t},\theta _{t})\big ), \end{aligned}$$

2.5c $$\begin{aligned} {\dot{e}}^{\epsilon }_{1,t}&= G_{1}(e^{\epsilon }_{t},\theta _{t}) \nonumber \\&\ \ \ +H_{1}(x+\epsilon \xi _{1},e^{\epsilon }_{t},\theta _{t})(1-P^{\epsilon }_{t})N^{\epsilon }_{t}+H_{1}(x+\epsilon \xi _{2},e^{\epsilon }_{t},\theta _{t})P^{\epsilon }_{t}N^{\epsilon }_{t}, \end{aligned}$$

2.5d $$\begin{aligned} e^{\epsilon }_{2,t}&= G_{2}(e^{\epsilon }_{t},\theta _{t}) \nonumber \\&\ \ \ +H_{2}(x+\epsilon \xi _{1},e^{\epsilon }_{t},\theta _{t})(1-P^{\epsilon }_{t})N^{\epsilon }_{t}+H_{2}(x+\epsilon \xi _{2},e^{\epsilon }_{t},\theta _{t})P^{\epsilon }_{t}N^{\epsilon }_{t}, \end{aligned}$$

2.5e $$\begin{aligned} {\dot{\theta }}_{t}&= A(\theta _{t})+B(\theta _{t}){\dot{W}}_{t}. \end{aligned}$$

Next, we unfold the dimorphic population dynamics of $x_{1}$ and $x_{2}$ based on the series expansion of model (2.5) in powers of $\epsilon$, and on time-scale separation. In the following analysis, we will see that $(N^{\epsilon }_{t},e^{\epsilon }_{t},\theta _{t})$ are fast variables with dynamics acted on *t*-timescale and $P^{\epsilon }_{t}$ is a slow variable with dynamics expounded on $\epsilon ^{i}t$-timescales for $i=1,2,\dots$.

#### Fast dynamics

For $\epsilon =0$, we get

2.6 $$\begin{aligned} \begin{aligned} {\dot{N}}^{0}_{t}&=f(x, e^{0}_{t},\theta _{t})N^{0}_{t}, \\ {\dot{P}}^{0}_{t}&=0, \\ {\dot{e}}^{0}_{1,t}&= G_{1}(e^{0}_{t},\theta _{t})+H_{1}(x,e^{0}_{t},\theta _{t})N^{0}_{t}, \\ e^{0}_{2,t}&= G_{2}(e^{0}_{t},\theta _{t})+H_{2}(x,e^{0}_{t},\theta _{t})N^{0}_{t}, \\ {\dot{\theta }}_{t}&= A(\theta _{t})+B(\theta _{t}){\dot{W}}_{t}. \end{aligned} \end{aligned}$$

A comparison of (2.6) and the dynamics of the reference monomorphic system $(x,n_{t},e_{t},\theta _{t})$ shows that $(N^{0}_{t},e^{0}_{t},\theta _{t})$ has the same dynamics as $(n_{t},e_{t},\theta _{t})$. Assume that $(x,n_{t},e_{t},\theta _{t})$ is a resident system with associated invariant probability measures $\mu$. Then, in the limit $\epsilon =0$, $(N^{\epsilon }_{t},e^{\epsilon }_{t},\theta _{t})$ enter the dynamics of the observables through $\mu$, and $P^{\epsilon }_{t}$ becomes irrelevant. Thus, (2.5) is a stochastic fast-slow system with fast variables $(N^{\epsilon }_{t},e^{\epsilon }_{t},\theta _{t})$ and slow variable $P^{\epsilon }_{t}$.

For positive but small $\epsilon$, the trajectories of $(N^{\epsilon }_{t},P^{\epsilon }_{t},e^{\epsilon }_{t},\theta _{t})$ can be viewed as a small variation of that of $(N^{0}_{t},P^{0}_{t},e^{0}_{t},\theta _{t})$ on *t*-timescale. Under some mild assumptions, the dynamics of (2.6) are arbitrarily good approximation of the dynamics of (2.5) in terms of conditional expectations when $\epsilon \rightarrow 0$ (ref to “Appendix B”).

#### Slow dynamics on $\epsilon t$-timescale

To study the dynamics of the relative population size on slow timescales, we have to take account of the high-order terms in the series expansion.

Consider the first-order term in the series expansion of the right side of (2.5b) and let $t_{1}=\epsilon t$, we have

2.7 $$\begin{aligned} {\dot{P}}^{\epsilon }_{t_{1}}=P^{\epsilon }_{t_{1}}(1-P^{\epsilon }_{t_{1}})f^{(1,0)}(x,e_{t_{1}/\epsilon },\theta _{t_{1}/\epsilon })^{\top }(\xi _{2}-\xi _{1})+\mathcal {O}(\epsilon ). \end{aligned}$$

Here the term $e^{0}_{t_{1}/\epsilon }$ in the series expansion has been replaced by $e_{t_{1}/\epsilon }$ because they have the same dynamics on *t*-timescale (see the interpretation in Sect. 2.5.1). Notice that $f^{(1,0)}(x,e,\theta )^{\top }$ becomes a row vector if *x* is a multi-dimensional strategy. Since $(x,n_{t},e_{t},\theta _{t})$ is assumed to be a resident system with associated invariant probability measures $\mu$ satisfying $\mu ({\mathcal {Z}}\setminus {\mathcal {Z}}_{0})=1$, we can apply Birkhoff’s ergodic theorem to an observation $f^{(1,0)}$ of $(x,n_{t},e_{t},\theta _{t})$, i.e., we have

2.8 $$\begin{aligned} \begin{aligned} \lim _{T\rightarrow +\infty }\frac{1}{T}\int _{0}^{T}f^{(1,0)}(x,e_{s},\theta _{s})^{\top }ds&= \int _{{\mathcal {Z}}\setminus {\mathcal {Z}}_{0}}f^{(1,0)}(x,e,\theta )^{\top }\mu (de,d\theta ) \\&= \partial _{y}{\mathcal {S}}_{x}(y)|_{y=x} \end{aligned} \end{aligned}$$

with probability one and for $\mu$-almost every $Z_{0}=z\in {\mathcal {Z}}\setminus {\mathcal {Z}}_{0}$. Here $\partial _{y}{\mathcal {S}}_{x}(y)|_{y=x}$ is so called the *selection gradient* at the reference strategy *x*. A strategy *x* is an *evolutionarily singular strategy* if

$$\begin{aligned} \partial _{y}{\mathcal {S}}_{x}(y)|_{y=x}=0. \end{aligned}$$

(ref to Metz et al. 1996; Geritz et al. 1997, 1998, 1999).

Throughout this section, assuming that

$$\begin{aligned} \partial _{y}{\mathcal {S}}_{x}(y)|_{y=x}(\xi _{2}-\xi _{1})\ne 0. \end{aligned}$$

Consider the following deterministic system

2.9 $$\begin{aligned} \dot{\bar{P}}_{t_{1}}=\bar{P}_{t_{1}}(1-\bar{P}_{t_{1}})\partial _{y}{\mathcal {S}}_{x}(y)|_{y=x}(\xi _{2}-\xi _{1}). \end{aligned}$$

**Fig. 2:**
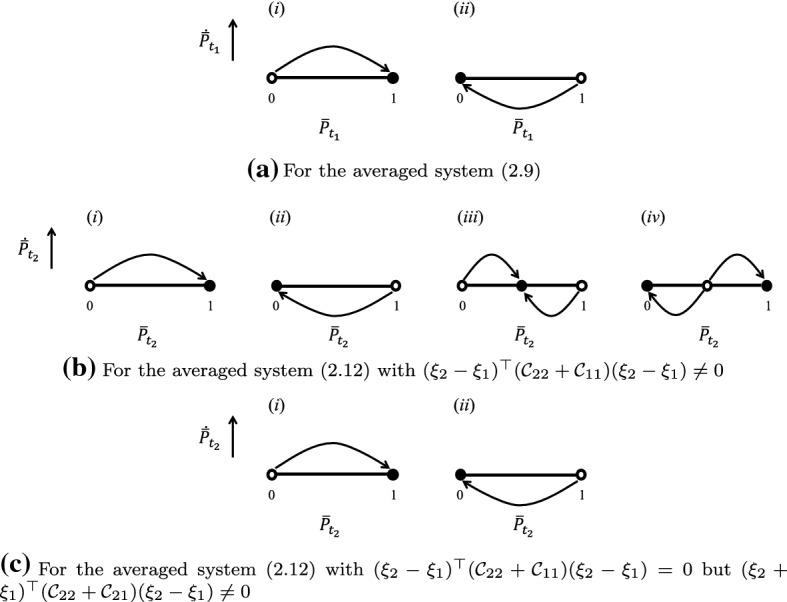
Equilibria and their stability of the associated averaged systems on different timescales, where “$\circ$” denotes an unstable equilibrium and “$\bullet$” denotes a stable equilibrium

From the averaging principle for fast-slow systems with stochastic and stationary fast dynamics, solutions of (2.7) are well approximated by solutions of (2.9) on every finite time interval when $\epsilon \rightarrow 0$, where (2.9) is so-called the *averaged system* with respect to (2.7) (ref to Freidlin and Wentzell (2012, Theorem 1.3 of Chapter 2 and Theorem 2.1 of Chapter 7), or Khas’minskii 1966; Arnold 2001; Kifer 2001; Liu and Krstic 2012). In the averaged system (2.9), there are exactly two equilibria:

$$\begin{aligned} 0,\ 1, \end{aligned}$$

with stabilities dominated by the signs of $\partial _{y}{\mathcal {S}}_{x}(y)|_{y=x}(\xi _{2}-\xi _{1})$. Figure 2a gives all generic cases. Under the stability of the averaged system (2.9), we show the property of solutions of (2.7) in arbitrarily long time intervals.

##### Lemma 1

Let $P^{\epsilon }_{0}=\bar{P}_{0}=p$ and $Z_{0}=z$. Assume that for a small $\sigma >0$,

$$\begin{aligned} \int _{{\mathcal {Z}}\setminus {\mathcal {Z}}_{0}}\big |f(y,e,\theta )\big |^{2}\mu (de,d\theta )<+\infty \ \textit{for all}\ y\in \big \{\tilde{x}\in {\mathcal {X}}:\Vert \tilde{x}-x\Vert \le \sigma \big \}, \end{aligned}$$

and $\lim _{t_{1}\rightarrow +\infty }\bar{P}_{t_{1}}=p^{*}$. Then for every $\delta \in (0,1)$, there exists a constant $T_{\delta }>0$ such that the solution of (2.7) satisfies

2.10 $$\begin{aligned} {\mathbb {P}}_{(p,z)}\bigg \{\lim _{\epsilon \rightarrow 0}\sup _{t_{1}\ge T_{\delta }}|P^{\epsilon }_{t_{1}}-p^{*}|\le \delta \bigg \}=1 \end{aligned}$$

for all $p\in [0,1]$ and for $\mu$-almost every $z\in {\mathcal {Z}}\setminus {\mathcal {Z}}_{0}$, where $p^{*}=0\ \text {or}\ 1$.

In words, for $\epsilon >0$ sufficiently small and at some later time, the trajectories of $P^{\epsilon }_{t_{1}}$ will remain $\delta$-close to the stable equilibrium of the averaged system (2.9) for arbitrarily long time with probability one.

##### Remark 1

In the hypotheses of Lemma 1, the boundedness ensures that the invasion fitness ${\mathcal {S}}_{x}(y)$ for a mutant strategy *y* close to the reference resident strategy *x* and the selection gradient $\partial _{y}{\mathcal {S}}_{x}(y)|_{y=x}$ are well defined, and it further guarantees the approximation of solutions of (2.7) and (2.9) in arbitrarily long time intervales.

Expansions of invasion fitnesses ${\mathcal {S}}_{x_{1}}(x_{2})$ and ${\mathcal {S}}_{x_{2}}(x_{1})$ up to the first order terms in $\epsilon$ give

2.11 $$\begin{aligned} {\mathcal {S}}_{x_{1}}(x_{2})=-\,{\mathcal {S}}_{x_{2}}(x_{1})=\epsilon \partial _{y}{\mathcal {S}}_{x}(y)|_{y=x}(\xi _{2}-\xi _{1}). \end{aligned}$$

Using (2.11), a stochastic analogy of the *invasion implies fixation theorem* of Geritz (2005, Proposition 1) immediately follows from Lemma 1, which is called as *invasion implies substitution theorem* in the present paper.

##### Theorem 1

(“Invasion implies substitution theorem”) Assume that the hypotheses of Lemma 1 are satisfied. Let the strategy pair $(x_{1},x_{2})$ and the reference strategy *x* are such that $\partial _{y}{\mathcal {S}}_{x}(y)|_{y=x}(\xi _{2}-\xi _{1})\ne 0$.

(i) If ${\mathcal {S}}_{x_{1}}(x_{2})>0$ and ${\mathcal {S}}_{x_{2}}(x_{1})<0$, then (2.10) holds for $p^{*}=1$.
(ii) If ${\mathcal {S}}_{x_{1}}(x_{2})<0$ and ${\mathcal {S}}_{x_{2}}(x_{1})>0$, then (2.10) holds for $p^{*}=0$.

For ${\mathcal {X}}\subset {\mathbb {R}}$, provided that $\partial _{y}{\mathcal {S}}_{x}(y)|_{y=x}\ne 0$ (i.e., *x* isn’t an evolutionarily singular strategy), the dimorphic population dynamics of $x_{1}$ and $x_{2}$ is completely determined by the invasion fitnesses. Since there are only two equilibria that are at the boundary for the averaged system (2.9), invasion implies substitution in terms that the dynamics of the relative population size satisfies (2.10). In other words, similar strategies cannot coexist when both of them are away from an evolutionarily singular strategy.

For ${\mathcal {X}}\subset {\mathbb {R}}^{d}$ with $d\ge 2$, geometrically, the deviation $\xi _{2}-\xi _{1}$ (which is equivalent to $x_{2}-x_{1}$) shall be away from the null-set of the linear form $\alpha \rightarrow \partial _{y}{\mathcal {S}}_{x}(y)|_{y=x}\alpha$ for all $\alpha \ne 0$ and $\partial _{y}{\mathcal {S}}_{x}(y)|_{y=x}$ non-vanishes, so that (2.9) is non-degenerate (i.e., the right side isn’t equal to zero). Consequently, in a multi-dimensional strategy space, invasion implies substitution provided that not only the two strategies are away from an evolutionarily singular strategy, but also their deviation isn’t orthogonal to the selection gradient at the reference strategy.

The averaged system (2.9) gives a geometric interpretation of the population dynamics of similar strategies in terms of their deviation and the selection gradient at a reference strategy, which indicates the sufficient condition of substitution of similar strategies. Thus, on $t_{1}$-timescale, any invader that can spread in a given resident system will ultimately oust the resident.

#### Slow dynamics on $\epsilon ^{2}t$-timescale

Once $\partial _{y}{\mathcal {S}}_{x}(y)|_{y=x}(\xi _{2}-\xi _{1})=0$, the right side of the averaged system (2.9) becomes zero. Then the high-order term $\mathcal {O}(\epsilon )$ in (2.7) plays a role on slower timescales.

Consider the second-order term in the series expansion of the right side of (2.5b) and let $t_{2}=\epsilon ^{2}t$, we have the associated averaged system:

2.12 $$\begin{aligned} \dot{\bar{P}}_{t_{2}}= & {} \bar{P}_{t_{2}}(1-\bar{P}_{t_{2}})\bigg ( (\xi _{2}+\xi _{1})^{\top }({\mathcal {C}}_{22}+{\mathcal {C}}_{21})(\xi _{2}-\xi _{1}) \nonumber \\&+\,\Big (\frac{1}{2}-\bar{P}_{t_{2}}\Big )(\xi _{2}-\xi _{1})^{\top }({\mathcal {C}}_{22} +{\mathcal {C}}_{11})(\xi _{2}-\xi _{1})\bigg ) \end{aligned}$$

with matrixes

2.13 $$\begin{aligned}&{\mathcal {C}}_{11}={\mathcal {C}}_{11}^{\top }=\frac{1}{2}\partial _{xx}{\mathcal {S}}_{x}(y)|_{y=x},\ {\mathcal {C}}_{12}={\mathcal {C}}_{21}^{\top }=\frac{1}{2}\partial _{xy}{\mathcal {S}}_{x}(y)|_{y=x}, \nonumber \\&{\mathcal {C}}_{21}={\mathcal {C}}_{12}^{\top }=\frac{1}{2}\partial _{yx}{\mathcal {S}}_{x}(y)|_{y=x},\ {\mathcal {C}}_{22}={\mathcal {C}}_{22}^{\top }=\frac{1}{2}\partial _{yy}{\mathcal {S}}_{x}(y)|_{y=x}. \end{aligned}$$

For the reader’s convenience, we give the detailed derivation of (2.12) in “Appendix D”. Notice that $\{{\mathcal {C}}_{ij}\}_{i,j=1,2}$ are matrices if *x* is a multi-dimensional strategy.

Throughout this section, assuming that

$$\begin{aligned} (\xi _{2}+\xi _{1})^{\top }({\mathcal {C}}_{22}+{\mathcal {C}}_{21})(\xi _{2}-\xi _{1})\ \text {and}\ (\xi _{2}-\xi _{1})^{\top }({\mathcal {C}}_{22}+{\mathcal {C}}_{11})(\xi _{2}-\xi _{1}) \end{aligned}$$

don’t vanish at the same time. If $(\xi _{2}-\xi _{1})^{\top }({\mathcal {C}}_{22}+{\mathcal {C}}_{11})(\xi _{2}-\xi _{1})\ne 0$, then the averaged system (2.12) has an interior equilibrium. In case of $(\xi _{2}-\xi _{1})^{\top }({\mathcal {C}}_{22}+{\mathcal {C}}_{11})(\xi _{2}-\xi _{1})=0$, only boundary equilibria exist. Figure 2b and c conclude all possible equilibria and their stability.

From the series expansions of invasion fitnesses ${\mathcal {S}}_{x_{1}}(x_{2})$ and ${\mathcal {S}}_{x_{2}}(x_{1})$, the second-order terms in $\epsilon$ give

2.14 $$\begin{aligned} \begin{aligned}&{\mathcal {S}}_{x_{1}}(x_{2})(1-\bar{P}_{t_{2}})-{\mathcal {S}}_{x_{2}}(x_{1})\bar{P}_{t_{2}} \\&\quad =\epsilon ^{2}\Big (\xi _{2}^{\top }{\mathcal {C}}_{22}\xi _{2}-\xi _{1}^{\top }{\mathcal {C}}_{22}\xi _{1}+2\big ((1-\bar{P}_{t_{2}})\xi _{1}+\bar{P}_{t_{2}}\xi _{2}\big )^{\top }{\mathcal {C}}_{21}(\xi _{2}-\xi _{1})\Big ) \\&\quad =\epsilon ^{2}\bigg ((\xi _{2}+\xi _{1})^{\top }({\mathcal {C}}_{22}+{\mathcal {C}}_{21})(\xi _{2}-\xi _{1}) \\&\qquad +\,\Big (\frac{1}{2}-\bar{P}_{t_{2}}\Big )(\xi _{2}-\xi _{1})^{\top }({\mathcal {C}}_{22}+{\mathcal {C}}_{11})(\xi _{2}-\xi _{1})\bigg ), \end{aligned} \end{aligned}$$

where the derivation of the last equality can be found in “Appendix D”. Using (2.14), the averaged system (2.12) have at most three equilibria in [0, 1]:

$$\begin{aligned} 0,\ \frac{{\mathcal {S}}_{x_{1}}(x_{2})}{{\mathcal {S}}_{x_{1}}(x_{2})+{\mathcal {S}}_{x_{2}}(x_{1})},\ 1, \end{aligned}$$

with stabilities dominated by the combination of signs of ${\mathcal {S}}_{x_{1}}(x_{2})$ and ${\mathcal {S}}_{x_{2}}(x_{1})$. Under the stability of the averaged system (2.12), we have a similar consequence as Lemma 1.

##### Lemma 2

Let $P^{\epsilon }_{0}=\bar{P}_{0}=p$ and $Z_{0}=z$. Assume that for a small $\sigma >0$,

$$\begin{aligned} \int _{{\mathcal {Z}}\setminus {\mathcal {Z}}_{0}}\big |f(y,e,\theta )\big |^{2}\mu (de,d\theta )<+\infty \ \textit{for all}\ y\in \big \{\tilde{x}\in {\mathcal {X}}:\Vert \tilde{x}-x\Vert \le \sigma \big \}. \end{aligned}$$

Then for every $\delta \in (0,1)$, there exists a constant $T_{\delta }>0$ such that

(i) if the averaged system (2.12) has a unique stable equilibrium, then the solution of (2.12) satisfies
  2.15 $$\begin{aligned} {\mathbb {P}}_{(p,z)}\bigg \{\lim _{\epsilon \rightarrow 0}\sup _{t_{2}\ge T_{\delta }}|P^{\epsilon }_{t_{2}}-p^{*}|\le \delta \bigg \}=1 \end{aligned}$$
  for all $p\in [0,1]$ and for $\mu$-almost every $z\in {\mathcal {Z}}\setminus {\mathcal {Z}}_{0}$, where $p^{*}=0$, or $\frac{{\mathcal {S}}_{x_{1}}(x_{2})}{{\mathcal {S}}_{x_{1}}(x_{2})+{\mathcal {S}}_{x_{2}}(x_{1})}$ or 1;
(ii) if the averaged system (2.12) has two stable equilibria (i.e., 0 and 1), then we have
  2.16 $$\begin{aligned} q^{0}_{(p,z)}+q^{1}_{(p,z)}=1 \end{aligned}$$
  for all $p\in [0,1]$ and for $\mu$-almost every $z\in {\mathcal {Z}}\setminus {\mathcal {Z}}_{0}$, where probabilities
  $$\begin{aligned} \begin{aligned} q^{0}_{(p,z)}&={\mathbb {P}}_{(p,z)}\bigg \{\lim _{\epsilon \rightarrow 0}\sup _{t_{2}\ge T_{\delta }}|P^{\epsilon }_{t_{2}}-0|\le \delta \bigg \}, \\ q^{1}_{(p,z)}&={\mathbb {P}}_{(p,z)}\bigg \{\lim _{\epsilon \rightarrow 0}\sup _{t_{2}\ge T_{\delta }}|P^{\epsilon }_{t_{2}}-1|\le \delta \bigg \}. \end{aligned} \end{aligned}$$

In words, for $\epsilon >0$ sufficiently small and at some later time, the trajectories of $P^{\epsilon }_{t_{2}}$ will remain $\delta$-close to the attractor (either a unique stable equilibrium or the bistable equilibria) of the averaged system (2.12) for arbitrarily long time with probability one.

##### Remark 2

In the hypotheses of Lemma 2, the boundedness ensures that (2.13) are well defined, and it further guarantees the approximation of solutions of $P^{\epsilon }_{t_{2}}$ and $\bar{P}_{t_{2}}$ in arbitrarily long time intervals. The boundedness is a technical requirement for Lemmas 1 and 2, which meets by many models.

Let $(\xi _{2}-\xi _{1})^{\top }({\mathcal {C}}_{22}+{\mathcal {C}}_{11})(\xi _{2}-\xi _{1})\ne 0$. Under the stability of the averaged system (2.12) with equivalent form (2.14), a stochastic analogy of the *classification theorem* of Geritz (2005, Proposition 2) immediately follows from Lemma 2.

##### Theorem 2

(“Classification theorem”) Assume that the hypotheses of Lemma 2 are satisfied. Let the strategy pair $(x_{1},x_{2})$ and the reference strategy *x* are such that $\partial _{y}{\mathcal {S}}_{x}(y)|_{y=x}(\xi _{2}-\xi _{1})=0$ but $(\xi _{2}-\xi _{1})^{\top }({\mathcal {C}}_{22}+{\mathcal {C}}_{11})(\xi _{2}-\xi _{1})\ne 0$.

(i) If ${\mathcal {S}}_{x_{1}}(x_{2})>0$ and ${\mathcal {S}}_{x_{2}}(x_{1})<0$, then (2.15) holds for $p^{*}=1$.
(ii) If ${\mathcal {S}}_{x_{1}}(x_{2})<0$ and ${\mathcal {S}}_{x_{2}}(x_{1})>0$, then (2.15) holds for $p^{*}=0$.
(iii) If ${\mathcal {S}}_{x_{1}}(x_{2})>0$ and ${\mathcal {S}}_{x_{2}}(x_{1})>0$, then (2.15) holds for $p^{*}=\frac{{\mathcal {S}}_{x_{1}}(x_{2})}{{\mathcal {S}}_{x_{1}}(x_{2})+{\mathcal {S}}_{x_{2}}(x_{1})}$.
(iv) If ${\mathcal {S}}_{x_{1}}(x_{2})<0$ and ${\mathcal {S}}_{x_{2}}(x_{1})<0$, then (2.16) holds.

Let $(\xi _{2}-\xi _{1})^{\top }({\mathcal {C}}_{22}+{\mathcal {C}}_{11})(\xi _{2}-\xi _{1})=0$. Then the averaged system (2.12) only has boundary equilibria. From the assertion (i) of Lemma 2, we have a special invasion-substitution theorem.

##### Theorem 3

(“Special invasion-substitution theorem”) Assume that the hypotheses of Lemma 2 are satisfied. Let the strategy pair $(x_{1},x_{2})$ and the reference strategy *x* are such that $\partial _{y}{\mathcal {S}}_{x}(y)|_{y=x}(\xi _{2}-\xi _{1})=0$ and $(\xi _{2}-\xi _{1})^{\top }({\mathcal {C}}_{22}+{\mathcal {C}}_{11})(\xi _{2}-\xi _{1})=0$ but $(\xi _{2}+\xi _{1})^{\top }({\mathcal {C}}_{22}+{\mathcal {C}}_{21})(\xi _{2}-\xi _{1})\ne 0$.

(i) If ${\mathcal {S}}_{x_{1}}(x_{2})>0$ and ${\mathcal {S}}_{x_{2}}(x_{1})<0$, then (2.15) for $p^{*}=1$.
(ii) If ${\mathcal {S}}_{x_{1}}(x_{2})<0$ and ${\mathcal {S}}_{x_{2}}(x_{1})>0$, then (2.15) for $p^{*}=0$.

**Fig. 3:**
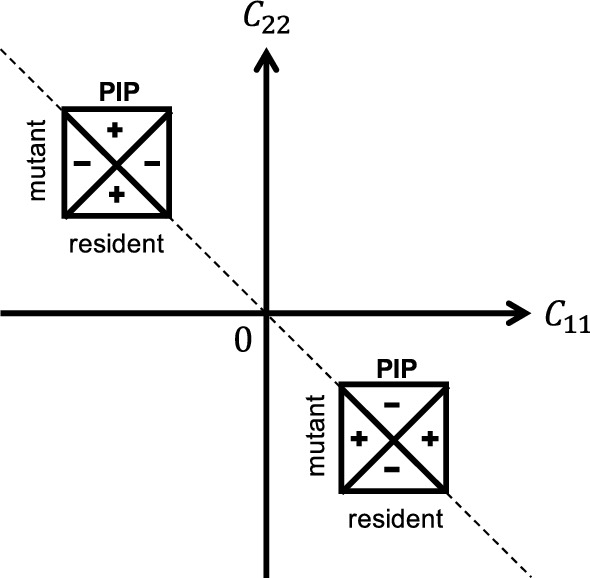
The geometric interpretation of the coexistence condition in a one-dimensional (or a one-dimensional parameterization of a multi-dimensional) strategy space. The “$+$” in the Pairwise Invasibility Plot (PIP) indicates the area where the mutant can invade the resident, and the “−” indicates the area where the mutant cannot invade

For ${\mathcal {X}}\subset {\mathbb {R}}$, Theorem 2 corresponds to the invasion dynamics of similar strategies close to a generic evolutionarily singular strategy that satisfies ${\mathcal {C}}_{22}+{\mathcal {C}}_{11}\ne 0$, while Theorem 3 corresponds to the invasion dynamics of similar strategies with $\xi _{1}+\xi _{2}\ne 0$ close to an evolutionarily singular strategy satisfying ${\mathcal {C}}_{22}+{\mathcal {C}}_{11}=0$ and ${\mathcal {C}}_{22}+{\mathcal {C}}_{21}\ne 0$. In the neighborhood of a generic evolutionarily singular strategy, the invasion dynamics is essentially “Lotka-Volterra”—dominance of one strategy, coexistence of two strategies, and mutual exclusion of two strategies—in sense that the relative population size remains $\delta$-close to the attractor of the averaged system for arbitrarily long time with probability one. In the neighborhood of an evolutionarily singular strategy satisfying ${\mathcal {C}}_{22}+{\mathcal {C}}_{11}=0$ and ${\mathcal {C}}_{22}+{\mathcal {C}}_{21}\ne 0$, only substitution happens for the invasion dynamics of any two similar but non-opposite strategies (i.e., $\xi _{1}+\xi _{2}\ne 0$). In Fig. 3, the dashed line indicates the scenario ${\mathcal {C}}_{22}+{\mathcal {C}}_{11}=0$ associated with the local configuration of the Pairwise Invasibility Plot (Matsuda 1985; van Tienderen and de Jong 1986). Invasion implies substitution if $({\mathcal {C}}_{11}, {\mathcal {C}}_{22})$ is on the dash line. Coexistence of similar strategies may occur if $({\mathcal {C}}_{11}, {\mathcal {C}}_{22})$ is away from the dash line.

For ${\mathcal {X}}\subset {\mathbb {R}}^{d}$ with $d\ge 2$, the hypothesis $(\xi _{2}-\xi _{1})^{\top }({\mathcal {C}}_{22}+{\mathcal {C}}_{11})(\xi _{2}-\xi _{1})\ne 0$ in Theorem 2 means that the deviation $\xi _{2}-\xi _{1}$ shall be away from the null-set of the quadratic form $\alpha \mapsto \alpha ^{\top }({\mathcal {C}}_{22}+{\mathcal {C}}_{11})\alpha$ for all $\alpha \ne 0$. Apart from $\partial _{y}{\mathcal {S}}_{x}(y)|_{y=x}(\xi _{2}-\xi _{1})=0$, Theorem 3 requires that $(\xi _{2}-\xi _{1})^{\top }({\mathcal {C}}_{22}+{\mathcal {C}}_{11})(\xi _{2}-\xi _{1})=0$ and $(\xi _{2}+\xi _{1})^{\top }({\mathcal {C}}_{22}+{\mathcal {C}}_{21})(\xi _{2}-\xi _{1})\ne 0$. The correspondingly geometric interpretation is complicate. However, if let $\xi _{1}=0$ (i.e., $x_{1}=x$), the two hypotheses are equivalent to $\xi _{2}^{\top }({\mathcal {C}}_{22}+{\mathcal {C}}_{11})\xi _{2}=0$ and $\xi _{2}^{\top }({\mathcal {C}}_{22}+{\mathcal {C}}_{21})\xi _{2}\ne 0$. From a conservation law ${\mathcal {C}}_{11}+{\mathcal {C}}_{22}+{\mathcal {C}}_{21}+{\mathcal {C}}_{12}=0$ (ref to (d.9) in “Appendix D”), we have ${\mathcal {C}}_{22}+{\mathcal {C}}_{21}=\frac{1}{2}({\mathcal {C}}_{22}-{\mathcal {C}}_{11})+\frac{1}{2}({\mathcal {C}}_{21}-{\mathcal {C}}_{12})$ where ${\mathcal {C}}_{22}-{\mathcal {C}}_{11}$ is symmetric and ${\mathcal {C}}_{21}-{\mathcal {C}}_{12}$ is skew-symmetric. Then by the property of skew-symmetric matrices, $\alpha ^{\top }({\mathcal {C}}_{21}-{\mathcal {C}}_{12})\alpha =0$ for all $\alpha$, we obtain that $\xi _{2}^{\top }({\mathcal {C}}_{22}+{\mathcal {C}}_{21})\xi _{2}=\frac{1}{2}\xi _{2}^{\top }({\mathcal {C}}_{22}-{\mathcal {C}}_{11})\xi _{2}$. Thus, if $\xi _{2}\ne 0$ is such that $\xi _{2}^{\top }({\mathcal {C}}_{22}+{\mathcal {C}}_{11})\xi _{2}=0$ but $\xi _{2}^{\top }C_{11}\xi _{2}\ne 0$ and $\xi _{2}^{\top }C_{22}\xi _{2}\ne 0$, then $\xi _{2}^{\top }({\mathcal {C}}_{22}+{\mathcal {C}}_{21})\xi _{2}\ne 0$ is readily satisfied.

##### Remark 3

Once $(\xi _{2}-\xi _{1})^{\top }({\mathcal {C}}_{22}+{\mathcal {C}}_{11})(\xi _{2}-\xi _{1})=0$ and $(\xi _{2}+\xi _{1})^{\top }({\mathcal {C}}_{22}+{\mathcal {C}}_{21})(\xi _{2}-\xi _{1})=0$, the higher order terms in $\epsilon$ of the series expansion are imperative to reveal the invasion dynamics of similar strategies. The degeneracy of a higher degree opens the possibility of unprotected coexistence for two strategies (ref to Priklopil (2012) and Dercole and Geritz (2016) for population models with point equilibria).

## Structured population models

In this section, we illustrate how to generalize the results of the unstructured population models to a class of structured population models.

Consider a structured population of strategies $x_{1},\dots ,x_{k}\in {\mathcal {X}}$ with corresponding sizes $n_{1,t},\dots ,n_{k,t}\in {\mathcal {N}}$ at time *t* where $n_{i,t}=(n^{1}_{i,t},\dots ,n^{\ell }_{i,t})^{\top }$ for $\ell \ge 2$ and for $i=1,\dots ,k$. Under the same considerations in Sect. 2.1, our polymorphic structured population model is

3.1 $$\begin{aligned} \begin{aligned} {\dot{n}}_{i,t}&= F(x_{i},e_{t},\theta _{t})n_{i,t},\ \ i=1,\dots ,k \\ {\dot{e}}_{1,t}&= G_{1}(e_{t},\theta _{t})+\sum _{j}H_{1}(x_{j},e_{t},\theta _{t})n_{j,t}, \\ e_{2,t}&= G_{2}(e_{t},\theta _{t})+\sum _{j}H_{2}(x_{j},e_{t},\theta _{t})n_{j,t}, \\ {\dot{\theta }}_{t}&= A(\theta _{t})+B(\theta _{t}){\dot{W}}_{t}, \end{aligned} \end{aligned}$$

where $F(x_{i},e_{t},\theta _{t})$ is a $\ell \times \ell$ matrix whose entries are transition rates between different states of individuals with strategy $x_{i}$ and depending on the current environmental condition $(e_{t},\theta _{t})$. Model (3.1) is an extension of the deterministic models used in Durinx et al. (2008) and Priklopil and Lehmann (2019), but now with an explicit formulation of environmental feedback and environmental stochasticity.

To place (3.1) into our framework, let $\Vert n_{i,t}\Vert _{1}$ be the total population size of $x_{i}$, and let $v_{i,t}$ be the proportion of each component in the population of $x_{i}$, i.e., for $i=1,\dots ,k$,

$$\begin{aligned} \Vert n_{i,t}\Vert _{1}=\sum _{j}n^{j}_{i,t},\ v_{i,t}=(v^{1}_{i,t},\cdots ,v^{\ell }_{i,t})^{\top } =\bigg (\frac{n^{1}_{i,t}}{\Vert n_{i,t}\Vert _{1}},\cdots , \frac{n^{\ell }_{i,t}}{\Vert n_{i,t}\Vert _{1}}\bigg )^{\top }. \end{aligned}$$

From (3.1), we obtain that for $i=1,\dots ,k$,

3.2 $$\begin{aligned} \begin{aligned} \Vert n_{i,t}\Vert _{1}^{\varvec{\cdot }}&= \mathbb {1}^{\top }F(x_{i},e_{t},\theta _{t})v_{i,t}\Vert n_{i,t}\Vert _{1}, \\ {\dot{v}}_{i,t}&= \big (F(x_{i},e_{t},\theta _{t})-\mathbb {1}^{\top }F(x_{i},e_{t},\theta _{t})v_{i,t}{\mathbb {I}}\big )v_{i,t}, \\ {\dot{e}}_{1,t}&= G_{1}(e_{t},\theta _{t})+\sum _{j}H_{1}(x_{j},e_{t},\theta _{t})v_{j,t}\Vert n_{j,t}\Vert _{1}, \\ e_{2,t}&= G_{2}(e_{t},\theta _{t})+\sum _{j}H_{2}(x_{j},e_{t},\theta _{t})v_{j,t}\Vert n_{j,t}\Vert _{1}, \\ {\dot{\theta }}_{t}&= A(\theta _{t})+B(\theta _{t}){\dot{W}}_{t}, \end{aligned} \end{aligned}$$

where $\mathbb {1}^{\top }=(1,\dots \,1)$ and ${\mathbb {I}}$ is the identity matrix. Although (3.2) cannot be written in the form of (2.1), the results in Sect. 2 still can be generalized to model (3.2) where population structures $v_{1,t},\dots ,v_{k,t}$ are viewed as auxiliary variables that lie in the space $\varDelta =\big \{v\in [0,1]^{\ell }:\Vert v\Vert _{1}=1\big \}$. Throughout this section, denote $Z_{t}=\big (\Vert n_{1,t}\Vert _{1},\dots ,\Vert n_{k,t}\Vert _{1},v_{1,t},\dots ,v_{k,t},e_{t},\theta _{t}\big )$. The state space of the dynamics of $Z_{t}$ is ${\mathcal {Z}}={\mathbb {R}}^{k}_{+}\times \varDelta ^{k}\times {\mathcal {E}}\times \varTheta$, the extinction set is ${\mathcal {Z}}_{0}=\big \{z\in {\mathcal {Z}}:\min _{i}\Vert n_{i}\Vert _{1}=0\big \}$, and the $\eta$-neighborhood of the extinction set is ${\mathcal {Z}}_{\eta }=\big \{z\in {\mathcal {Z}}:\min _{i}\Vert n_{i}\Vert _{1}\le \eta \big \}$. Like Assumption **A2**, the partial derivative $F^{(i,j)}$ requires to exit and to be locally Lipschitz continuous and measurable in $(e,\theta )$ for all strategies and for all nonnegative integers $i,j\le 2$.

Next, we focus on the analysis of dimorphic population dynamics of similar strategies. Like the discussion in Sect. 2.5, let $x_{1}=x+\epsilon \xi _{1}$, $x_{2}=x+\epsilon \xi _{2}\in {\mathcal {X}}$ with corresponding population sizes $n^{\epsilon }_{1,t}$, $n^{\epsilon }_{2,t}\in {\mathcal {N}}$ and population structures $v^{\epsilon }_{1,t}$, $v^{\epsilon }_{2,t}\in \varDelta$ at time *t*. Let $\big (x,\Vert n_{t}\Vert _{1},v_{t},e_{t},\theta _{t}\big )$ is the reference monomorphic resident system with invariant probability measure $\mu$. We now are interested in the dynamics of the total population size $N^{\epsilon }_{t}$ of $x_{1}$ and $x_{2}$ and the relative population size $P^{\epsilon }_{t}$ of $x_{2}$, where

$$\begin{aligned} N^{\epsilon }_{t}=\Vert n^{\epsilon }_{1,t}\Vert _{1}+\Vert n^{\epsilon }_{2,t}\Vert _{1},\ P^{\epsilon }_{t}=\frac{\Vert n^{\epsilon }_{2,t}\Vert _{1}}{N^{\epsilon }_{t}}. \end{aligned}$$

Expanding (3.2) around $\epsilon =0$ (i.e., $x_{1}=x_{2}=x$) and then taking $\epsilon =0$, we have

3.3a $$\begin{aligned} {\dot{N}}^{0}_{t}&=\mathbb {1}^{\top }F(x,e^{0}_{t},\theta _{t})\big (v^{0}_{2,t}P^{0}_{t}+v^{0}_{1,t}(1-P^{0}_{t})\big )N^{0}_{t}, \end{aligned}$$

3.3b $$\begin{aligned} {\dot{P}}^{0}_{t}&=P^{0}_{t}(1-P^{0}_{t})\mathbb {1}^{\top }F(x,e^{0}_{t},\theta _{t})(v^{0}_{2,t}-v^{0}_{1,t}). \end{aligned}$$

Recalling the analysis in Sect. 2.5.1, the difference here is that $P^{0}_{t}$ is not longer a constant on *t*-timescale. Instead, $P^{0}_{t}$ evolves with $N^{0}_{t}$ on *t*-timescale. The following we derive that (i) the dynamics of the monomorphic system $\big (\Vert n_{t}\Vert _{1},v_{t},e_{t},\theta _{t}\big )$ still represents the dynamics of $(N^{\epsilon }_{t},v^{\epsilon }_{i,t},e^{\epsilon }_{t},\theta _{t})$ on *t*-timescale when $\epsilon =0$ for $i=1,2$, and (ii) $P^{\epsilon }_{t}$ evolves on *t*-timescale but eventually returns to its initial state when $\epsilon =0$.

Denote

$$\begin{aligned} \bar{v}^{0}_{t}=v^{0}_{2,t}P^{0}_{t}+v^{0}_{1,t}(1-P^{0}_{t}), \end{aligned}$$

which is the averaged proportion of population structures $v^{0}_{1,t}$ and $v^{0}_{2,t}$ at time *t*. From (3.3a) and the dynamics of $(v^{0}_{1,t},v^{0}_{2,t},e^{0}_{t})$, we have

3.4 $$\begin{aligned} \begin{aligned} {\dot{N}}^{0}_{t}&=\mathbb {1}^{\top }F(x,e^{0}_{t},\theta _{t})\bar{v}^{0}_{t}N^{0}_{t}, \\ \dot{\bar{v}}^{0}_{t}&= \big (F(x,e^{0}_{t},\theta _{t})-\mathbb {1}^{\top }F(x,e^{0}_{t},\theta _{t})\bar{v}^{0}_{t}{\mathbb {I}}\big )\bar{v}^{0}_{t}, \\ {\dot{e}}^{0}_{1,t}&= G_{1}(e^{0}_{t},\theta _{t})+H_{1}(x,e^{0}_{t},\theta _{t})\bar{v}^{0}_{t}N^{0}_{t}, \\ e^{0}_{2,t}&= G_{2}(e^{0}_{t},\theta _{t})+H_{2}(x,e^{0}_{t},\theta _{t})\bar{v}^{0}_{t}N^{0}_{t}, \\ {\dot{\theta }}_{t}&= A(\theta _{t})+B(\theta _{t}){\dot{W}}_{t}. \end{aligned} \end{aligned}$$

It shows that $(N^{0}_{t},\bar{v}^{0}_{t},e^{0}_{t},\theta _{t})$ satisfies the same equations as $\big (\Vert n_{t}\Vert _{1},v_{t},e_{t},\theta _{t}\big )$. In words, the dynamics of $\big (\Vert n_{t}\Vert _{1},v_{t},e_{t},\theta _{t}\big )$ represents that of $(N^{\epsilon }_{t},\bar{v}^{\epsilon }_{t},e^{\epsilon }_{t},\theta _{t})$ on *t*-timescale when $\epsilon =0$. Turning to the dynamics of $v^{0}_{1,t}$ and $v^{0}_{2,t}$,

$$\begin{aligned} {\dot{v}}^{0}_{i,t}=\big (F(x,e^{0}_{t},\theta _{t})-\mathbb {1}^{\top }F(x,e^{0}_{t},\theta _{t})v^{0}_{i,t}{\mathbb {I}}\big )v^{0}_{i,t},\ \ i=1,2 \end{aligned}$$

with $e^{0}_{t}$ calculated from (3.4), we see that $v^{0}_{1,t}$ and $v^{0}_{2,t}$ satisfy the same equation as $\bar{v}^{0}_{t}$ (or, equivalently, $v_{t}$). Then $v^{0}_{1,t}$ and $v^{0}_{2,t}$ have the same dynamics as $\bar{v}^{0}_{t}$ (or, equivalently, $v_{t}$). Thus, we claim that $(N^{\epsilon }_{t},v^{\epsilon }_{i,t},e^{\epsilon }_{t},\theta _{t})$ has the same dynamics as $\big (\Vert n_{t}\Vert _{1},v_{t},e_{t},\theta _{t}\big )$ on *t*-timescale when $\epsilon =0$ for $i=1,2$.

Now we return to (3.3b), a straightforward calculation shows that

$$\begin{aligned} P^{0}_{t}=\Bigg (1+\frac{1-p}{p}\exp \bigg (\int _{0}^{t}\mathbb {1}^{\top }F(x,e^{0}_{\tau },\theta _{\tau })(v^{0}_{2,\tau }-v^{0}_{1,\tau })d\tau \bigg )\Bigg )^{-1} \end{aligned}$$

provided that the initial state is $(n,v_{1},v_{2},e,\theta ,p)$. Using Birkhoff’s ergodic theorem and the principle of selective neutrality of residents (i.e., zero growth rate of the reference resident *x*), we obtain

$$\begin{aligned} \lim _{t\rightarrow +\infty }P^{0}_{t}=p \end{aligned}$$

with probability one and for all $p\in [0,1]$ and for $\mu$-almost every $(n,v_{1},v_{2},e,\theta )$ (N.B., initial states $v_{1}$ and $v_{2}$ do not have to be identical). It means that $P^{0}_{t}$ will sufficiently close to its initial state some time later with probability one. Thus, we need consider the dynamics of $P^{\epsilon }_{t}$ on slow timescales to revel the dimorphic population dynamics of $x_{1}$ and $x_{2}$.

The remaining generalization of our theorems to model (3.2) can be done by using of arguments similar to Sects. 2.5.2 and 2.5.3.

## Applications

To illustrate the utility of our theorems, we apply them to four concrete examples. The first example we consider the evolving bacteria in a chemostat model. We will see that substitution is the unique outcome after an invasion event. The second example we consider a Lotka-Volterra competition model. Theorems 1, 2 and 3 are applied to predict the population dynamical outcomes of an invasion event when the strategy pairs satisfy the corresponding hypotheses of these theorems. The third example we consider a structured SIRS epidemic model, which is designed to illustrate how a concrete structured population model can be reformulated into our framework. In the last example, we consider the evolution of timidity of the prey in a predator-prey model, which is designed to demonstrate that our results are applicable to the evolution with the non-equilibrium resident dynamics.

In these four examples, we start from a monomorphic population with a certain strategy. To verify the existence of an invariant probability measure for a resident system, one might consider the tightness of one of the following two families of measures:

- the statistics associated with a single realization of process $\{Z_{t}\}_{t\ge 0}$, i.e. measure
  $$\begin{aligned} \varPi ^{z}_{t}(\cdot )=\frac{1}{t}\int _{0}^{t}\delta _{Z_{s}}(\cdot )ds \end{aligned}$$
  with a Dirac measure $\delta _{Z_{s}}$ at $Z_{s}$ (ref to the argument presented in Schreiber et al. (2011) or Benaïm (2018));
- the statistics associated with transition probabilities $\{{\mathcal {P}}_{t}\}_{t\ge 0}$ of process $\{Z_{t}\}_{t\ge 0}$, i.e. measure
  $$\begin{aligned} \pi ^{z}_{t}(\cdot )=\frac{1}{t}\int _{0}^{t}{\mathcal {P}}_{s}(z,\cdot )ds \end{aligned}$$
  which is a common tool in the literature on Markov process (see e.g. Arnold and Kliemann 1983, Proposition 3.15).

Different ways to study the existence of invariant probability measures give different interpretations for the asymptotic behavior of process $\{Z_{t}\}_{t\ge 0}$. Assume that the limit exists as time goes to infinity, the (a) gives the long-term frequency of a typical realization of process $\{Z_{t}\}_{t\ge 0}$ visiting a particular configuration. Since a transition probability corresponds to the frequency of observing a particular event across many realizations of process $\{Z_{t}\}_{t\ge 0}$, the (b) gives the long-term frequency of probabilities for process visiting a particular configuration. Following the strategies of proofs of Benaïm (2018, Theorem 4.4), Schreiber et al. (2011) and Arnold and Kliemann (1983, Proposition 3.15), one can show that all weak$^{\star }$ limit points of $\varPi ^{z}_{t}$ and $\pi ^{z}_{t}$ are candidate $\mu$ of Definition 1.

**Fig. 4:**
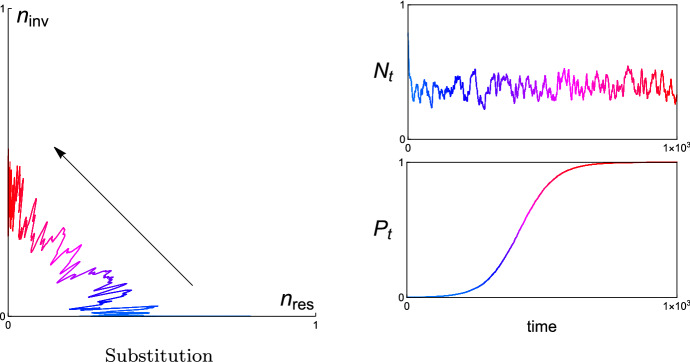
A simulated population trajectory in the phase plane and associated dynamics of total concentration $N_{t}=n_{res,t}+n_{inv,t}$ and relative concentration $P_{t}=n_{inv,t}/N_{t}$ for a resident bacteria with strategy $x=(2,0.8,0.095)$ and an invader bacteria with strategy $y=(1.99,0.85,0.09)$. The corresponding invasion fitnesses ${\mathcal {S}}_{x}(y)= 0.0161516> 0$ and ${\mathcal {S}}_{y}(x)=-\,0.0102779 < 0$. Parameter values $D=0.1$, $\rho _{1}=1$, $\rho _{2}=0.5$, $a=b=1$

In each example, we present the simulated trajectories of (i) the resident and invader population in the corresponding phase plane, (ii) the total population size $N_{t}$, and (iii) the relative population size $P_{t}$ of the invader. The numerical analysis shows that $N_{t}$ varies stochastically and dramatically in time, while $P_{t}$ changes slowly and asymptotically in time. Since we don’t perform the complete time-scale separation in numerical simulations, one can find that there are some small fluctuations remain in the simulated trajectories of $P_{t}$ in Figs. 4, 6, 8, and 10 for different examples, respectively. With complete time-scale separation (i.e., $\epsilon \rightarrow 0$), the trajectories of $P_{t}$ will be smooth.

### Chemostat model

Consider the evolving bacteria with strategy $x=(\beta ,\gamma ,\delta )\in {\mathbb {R}}^{3}_{+}:={\mathcal {X}}$ and concentration $n_{t}$ at time *t* in a chemostat model, in which the dynamics is given by

4.1 $$\begin{aligned} \begin{aligned} {\dot{R}}_{t}&=D(R^{in}_{t}-R_{t})-\beta R_{t}n_{t}, \\ {\dot{n}}_{t}&=\gamma \beta R_{t}n_{t}-(\delta +D)n_{t},\ \ \end{aligned} \end{aligned}$$

where $R_{t}$ is the concentration of nutrient at time *t*. Let $R^{in}_{t}$ be the time-varying concentration of nutrient at the input. Positive constants *D*, $\beta$, $\gamma$ and $\delta$ are dilution rate, nutrient uptake rate, conversion efficiency and death rate of bacteria, respectively. In this model, we assume

$$\begin{aligned} R^{in}_{t}=\rho _{1}-\rho _{2}\frac{2\theta _{t}}{1+\theta ^{2}_{t}} \end{aligned}$$

with constants $\rho _{1}>\rho _{2}>0$ and $\{\theta _{t}\}_{t\geqslant 0}$ is the stationary Ornstein-Uhlenbeck process generated by a linear stochastic differential equation

4.2 $$\begin{aligned} {\dot{\theta }}_{t}=-\,a\theta _{t}+b{\dot{W}}_{t},\ \ \theta _{0}=0, \end{aligned}$$

with positive constants *a* and *b*. Then $R^{in}_{t}$ takes value in the interval $[\rho _{1}-\rho _{2},\rho _{1}+\rho _{2}]$ for all time *t* and tends to peaks around $\rho _{1}\pm \rho _{2}$, which is a random switching scenario. Different ways that an unbounded noise induces bounded fluctuations on model parameters refer to d’Onofrio (2013) and Caraballo and Han (2016).

Denote

$$\begin{aligned} \begin{aligned} e_{t}=(e_{1,t},e_{2,t})&= (R_{t},0), \\ f(x,e_{t},\theta _{t})&= \gamma \beta e_{1,t}-\delta -D, \\ G_{1}(e_{t},\theta _{t})&= D(R^{in}_{t}-e_{1,t}), \\ H_{1}(x,e_{t},\theta _{t})&= \beta e_{1,t}. \end{aligned} \end{aligned}$$

Then (4.1) can be written as the general form (2.1). From the boundedness of $R^{in}_{t}$, one can show that $R_{t}\le \rho _{1}+\rho _{2}$ and $n_{t}\le \frac{\gamma \beta (\rho _{1}+\rho _{2})}{\delta +D}$ for *t* sufficiently large. Thus, the state space of the dynamics of $(n_{t},e_{t},\theta _{t})$ is ${\mathcal {Z}}=\big [0,\frac{\gamma \beta (\rho _{1}+\rho _{2})}{\delta +D}\big ]\times [0,\rho _{1}+\rho _{2}]\times \{0\}\times {\mathbb {R}}$. The partial derivatives $f^{(i,j)}$, $G^{(i,j)}_{1}$ and $H^{(i,j)}_{1}$ exist for all strategies $x\in {\mathcal {X}}$ and for all nonnegative integers $i,j\le 2$, which satisfy Assumption **A2**.

For bacteria *x*, its virgin environment $R^{vir}_{t}$ is generated by

$$\begin{aligned} {\dot{R}}^{vir}_{t}=D(R^{in}_{t}-R^{vir}_{t}). \end{aligned}$$

The boundedness of $R^{in}_{t}$ and the Feller property ensure the existence of invariant probability measures for process $\{R^{vir}_{t},\theta _{t}\}_{t\ge 0}$ with support $[\rho _{1}-\rho _{2},\rho _{1}+\rho _{2}]\times {\mathbb {R}}\subset {\mathcal {Z}}_{0}$ and expectation ${\mathbb {E}}[R^{vir}]={\mathbb {E}}[R^{in}]=\rho _{1}$. Bacteria *x* can invade the virgin environment $R^{vir}$ if

4.3 $$\begin{aligned} \gamma \beta {\mathbb {E}}[R^{vir}]-\delta -D=\gamma \beta \rho _{1}-\delta -D>0. \end{aligned}$$

Likewise, the boundedness of $(n_{t},R_{t})$ and the Feller property ensure the existence of invariant probability measures for process $\{n_{t},R_{t},\theta _{t}\}_{t\ge 0}$, denoted by $\mu$. One hand, by Poincaré recurrence theorem and the boundedness of $n_{t}$, we obtain that $\lambda _{x}(\mu )=0$ for all $\mu$ with $\mu ({\mathcal {Z}}\setminus {\mathcal {Z}}_{0})=1$ (ref to Schreiber et al. 2011, assertion (iii) of Proposition 1). On the other hand, since ${\mathcal {Z}}\setminus {\mathcal {Z}}_{0}$ and ${\mathcal {Z}}_{0}$ are invariant, there exists $\alpha \in (0,1]$ such that $\mu =(1-\alpha )\mu _{0}+\alpha \mu _{1}$ where $\mu _{0}$ is an invariant probability measure with $\mu _{0}({\mathcal {Z}}_{0})=1$ and $\mu _{1}$ is an invariant probability measure with $\mu _{1}({\mathcal {Z}}\setminus {\mathcal {Z}}_{0})=1$. Notice that $\lambda _{x}(\mu )\le 0$ due to the boundedness of $n_{t}$. From this fact and $\lambda _{x}(\mu _{1})=0$, it follows that $(1-\alpha )\lambda _{x}(\mu _{0})\le 0$. By (4.3) (i.e., $\lambda _{x}(\mu _{0})>0$), we obtain that $\alpha =1$. Thus, all $\mu$ satisfy $\mu ({\mathcal {Z}}\setminus {\mathcal {Z}}_{0})=1$, provided that (4.3) holds. Using (4.3) and arguments similar to the proof of Benaïm (2018, Theorem 4.4), we further can show that for all $\varepsilon >0$, there exists a $\eta >0$ such that $\mu ({\mathcal {Z}}_{\eta })\le \varepsilon$ for every $\mu$ with $\mu ({\mathcal {Z}}\setminus {\mathcal {Z}}_{0})=1$. From Definition 1, we thus claim that bacteria *x* is non-growing on the long-run and stochastically persistent.

Once bacteria *x* becomes a resident, the invasion fitness of an initially rare mutant $y=(\beta ^{\prime },\gamma ^{\prime },\delta ^{\prime })$ in resident *x* is

$$\begin{aligned} {\mathcal {S}}_{x}(y)=\gamma ^{\prime }\beta ^{\prime }{\mathbb {E}}[R]-\delta ^{\prime }-D. \end{aligned}$$

For similar strategies (*x*, *y*) satisfying $\partial _{y}{\mathcal {S}}_{x}(y)|_{y=x}(y-x)\ne 0$, Theorem 1 can be employed to predict the invasion dynamics. Instead we apply Theorems 2 or 3 if $\partial _{y}{\mathcal {S}}_{x}(y)|_{y=x}(y-x)=0$. However, we will see that substitution is the unique outcome after an invasion event whatever strategy pairs are. In fact, from (2.4), we get that ${\mathbb {E}}[R]=\frac{\delta +D}{\gamma \beta }$. Hence, invasion happens if and only if $\frac{\delta ^{\prime }+D}{\gamma ^{\prime }\beta ^{\prime }}<\frac{\delta +D}{\gamma \beta }$. It further implies that for all *x*, $y\in {\mathcal {X}}$, ${\mathcal {S}}_{y}(x)<0$ if ${\mathcal {S}}_{x}(y)>0$.

Figure 4 shows how a specific population trajectory develops from $n_{res}$-axis to $n_{inv}$-axis in the phase plane provided that $x=(2,0.8,0.095)$ and $y=(1.99,0.85,0.09)$ with ${\mathcal {S}}_{x}(y)> 0$ and ${\mathcal {S}}_{y}(x)< 0$. The initially rare bacteria *y* will actually take over the bacteria *x* and becomes the new resident. The total concentration $N_{t}$ of the resident *x* and the invader *y* varies stochastically and dramatically in time. However, the relative concentration $P_{t}$ of the invader *y* changes slowly in time and asymptotically closes to 1. The dynamics of $P_{t}$ illustrates that the resident *x* is substituted by the invader *y* eventually.

### Lotka–Volterra competition model

Consider a stochastic Lotka-Volterra competition model

4.4 $$\begin{aligned} {\dot{n}}_{i,t}=\tilde{r}_{t}(x_{i})n_{i,t} \bigg (1-\frac{\sum _{j}\alpha (x_{i},x_{j})n_{j,t}}{\widetilde{K}_{t}(x_{i})}\bigg ),\ \ i=1,\dots ,k \end{aligned}$$

where $n_{i,t}$ is the population size of strategy $x_{i}\in {\mathbb {R}}:={\mathcal {X}}$. Assume that the intrinsic growth rate and the carrying capacity of the *i*-th species are influenced by an external factor $\theta _{t}$ such that they fluctuate around strategy-related values. For simplicity, let

$$\begin{aligned} \begin{aligned} \tilde{r}_{t}(x_{i})&= r(x_{i})\exp (\rho _{1}\theta _{t})=\exp (-d_{1}x^{2}_{i}+\rho _{1}\theta _{t}), \\ \widetilde{K}_{t}(x_{i})&= K(x_{i})\exp (\rho _{2}\theta _{t})=\exp (-d_{2}x^{2}_{i}+\rho _{2}\theta _{t}) \end{aligned} \end{aligned}$$

where $d_{1}$, $d_{2}$, $\rho _{1}$ and $\rho _{2}$ are scaling parameters and where the $\{\theta _{t}\}_{t\ge 0}$ is a stationary Ornstein-Uhlenbeck process generated by (4.2). Then the expected growth rate and the expected carrying capacity of the *i*-th species are $r(x_{i})\exp \big (\frac{\rho ^{2}_{1}b^{2}}{2a}\big )$ and $K(x_{i})\exp \big (\frac{\rho ^{2}_{2}b^{2}}{2a}\big )$, respectively. Let the competitive coefficient between strategy $x_{i}$ and $x_{j}$ be of the form

$$\begin{aligned} \begin{aligned}&\alpha (x_{i},x_{j}) \\&=\big (1-(x_{j}-x_{i})(c_{0}+c_{1}x^{2}_{i}+c_{2}x_{i}x_{j}+c_{3}x^{2}_{j}) \exp (c_{4}x^{2}_{j})\big )\exp \big (c_{5}(x^{2}_{i}-x^{2}_{j})\big ), \end{aligned} \end{aligned}$$

where $\alpha (x_{i},x_{i})=1$ for all *i*.

Let ${\mathcal {E}}$ be the set of all functions $e:{\mathcal {X}}\mapsto {\mathbb {R}}^{2}$ of the form

$$\begin{aligned} e_{t}(\cdot )=(e_{1,t},e_{2,t})(\cdot )=\bigg (0,\ \sum _{j}\alpha (\cdot ,x_{j})n_{j,t}\bigg ) \end{aligned}$$

for $n_{j,t}\ge 0$ and $x_{j}\in {\mathcal {X}}$. Since the competition is modelled as a direct interaction, we can define the per-capita environmental impact $H_{2}:{\mathcal {X}}\times {\mathcal {E}}\times {\mathbb {R}}\mapsto {\mathbb {R}}$ by

$$\begin{aligned} H_{2}(x_{j},e_{t},\theta )(\cdot )=\alpha (\cdot ,x_{j}) \end{aligned}$$

which is the competitive impact of a single individual with strategy $x_{j}$ on its competitor with any strategy. Since the competitor’s strategy has infinitely many choices, $e_{2,t}$ has infinite dimensions. In addition, define the map $G_{2}:{\mathcal {E}}\times {\mathbb {R}}\mapsto {\mathbb {R}}$ by

$$\begin{aligned} G_{2}(e_{t},\theta )=0. \end{aligned}$$

Further, define the map $f:{\mathcal {X}}\times {\mathcal {E}}\times {\mathbb {R}}\mapsto {\mathbb {R}}$ by

$$\begin{aligned} f\big (x_{i},e_{t}(x_{i}),\theta \big )=\tilde{r}(x_{i}) \bigg (1-\frac{e_{2,t}(x_{i})}{\widetilde{K}(x_{i})}\bigg ). \end{aligned}$$

Thus, (4.4) can be written as the general form (2.1). Furthermore, the state space of the dynamics of $(n_{1,t},\dots ,n_{k,t},e_{t},\theta _{t})$ is ${\mathcal {Z}}={\mathbb {R}}^{k}_{+}\times {\mathcal {E}}\times {\mathbb {R}}$. The partial derivatives $f^{(i,j)}$, $G^{(i,j)}_{2}$ and $H^{(i,j)}_{2}$ exist for all strategies and for all nonnegative integers $i,j\le 2$, which satisfy Assumption **A2**.

**Fig. 5:**
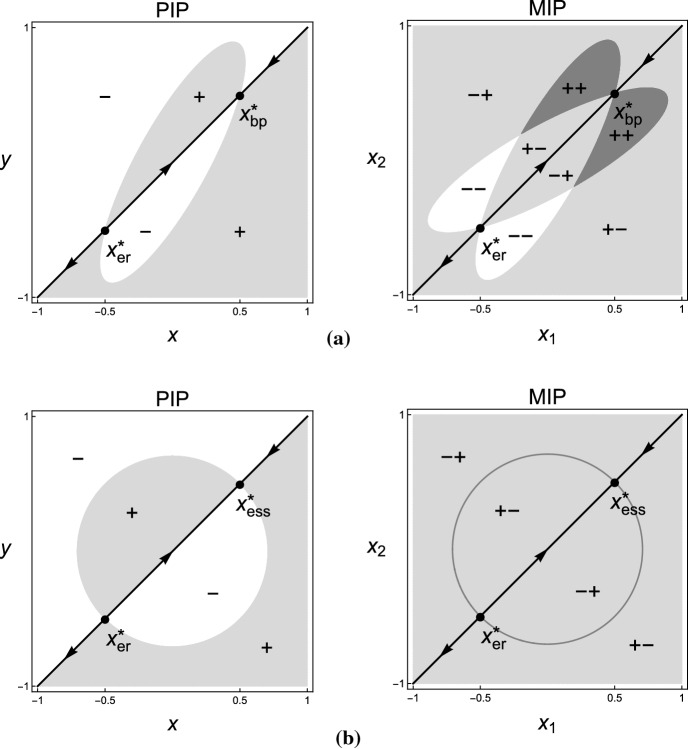
Pairwise invasibility plot (PIP) and corresponding Mutual invasibility plot (MIP) for the invasion fitness (4.5) with parameter values $d_{1}=1$, $d_{2}=0.5$, $\rho _{1}=1$, $\rho _{2}=0.8$, $a=b=1$, and $(c_{0},c_{1},c_{2},c_{3},c_{4},c_{5})=$ **(a)** $(1,-11,11,-4,1,0.5)$ and **(b)** $(1,-2,0,-2,1,0.5)$. White area in PIP: *y* cannot invade *x*; light gray area in PIP: *y* can invade *x*; white area in MIP: $x_{1}$ and $x_{2}$ cannot invade each other; light gray area in MIP: one of $x_{1}$ and $x_{2}$ can invader the other but not *vice versa*; gray area in MIP: $x_{1}$ and $x_{2}$ can invade each other; “$+$” and “−”: signs of the invasion fitness for given strategy pairs; arrows: the direction of monomorphic evolution

We now start from a monomorphic population of strategy *x* with size $n_{t}$ at time *t*. Similar to the previous example, one can show that species *x* successfully establishes with population dynamics satisfying Definition 1. The only difference that should be pointed out is that the population is unbounded in this example. Instead the population is ultimately bounded in mean (Miyahara 1972, 1973), i.e., $\limsup _{t\rightarrow +\infty }{\mathbb {E}}[n_{t}]\le {\mathbb {E}} \big [\limsup _{t\rightarrow +\infty }n_{t}\big ]=K(x){\mathbb {E}} \big [\limsup _{t\rightarrow +\infty }\exp (\rho _{2}\theta _{t})\big ]=K(x)<+\infty$ for all $x\in {\mathcal {X}}$. Further, this stochastic boundedness combined with the Feller property guarantees the existence of invariant probability measures for process $\{n_{t},\theta _{t}\}_{t\ge 0}$. Now, from (4.4), the invasion fitness of an initially rare mutant *y* in resident *x* is given by

4.5 $$\begin{aligned} {\mathcal {S}}_{x}(y)=r(y)\exp \bigg (\frac{\rho ^{2}_{1}\sigma ^{2}}{2a}\bigg ) \bigg (1-\frac{\alpha (y,x)K(x)}{K(y)}\bigg ). \end{aligned}$$

Here we have used the principle of selective neutrality of residents (i.e., ${\mathcal {S}}_{x}(x)=0$ for all $x\in {\mathcal {X}}$) to derive (4.5).

**Fig. 6:**
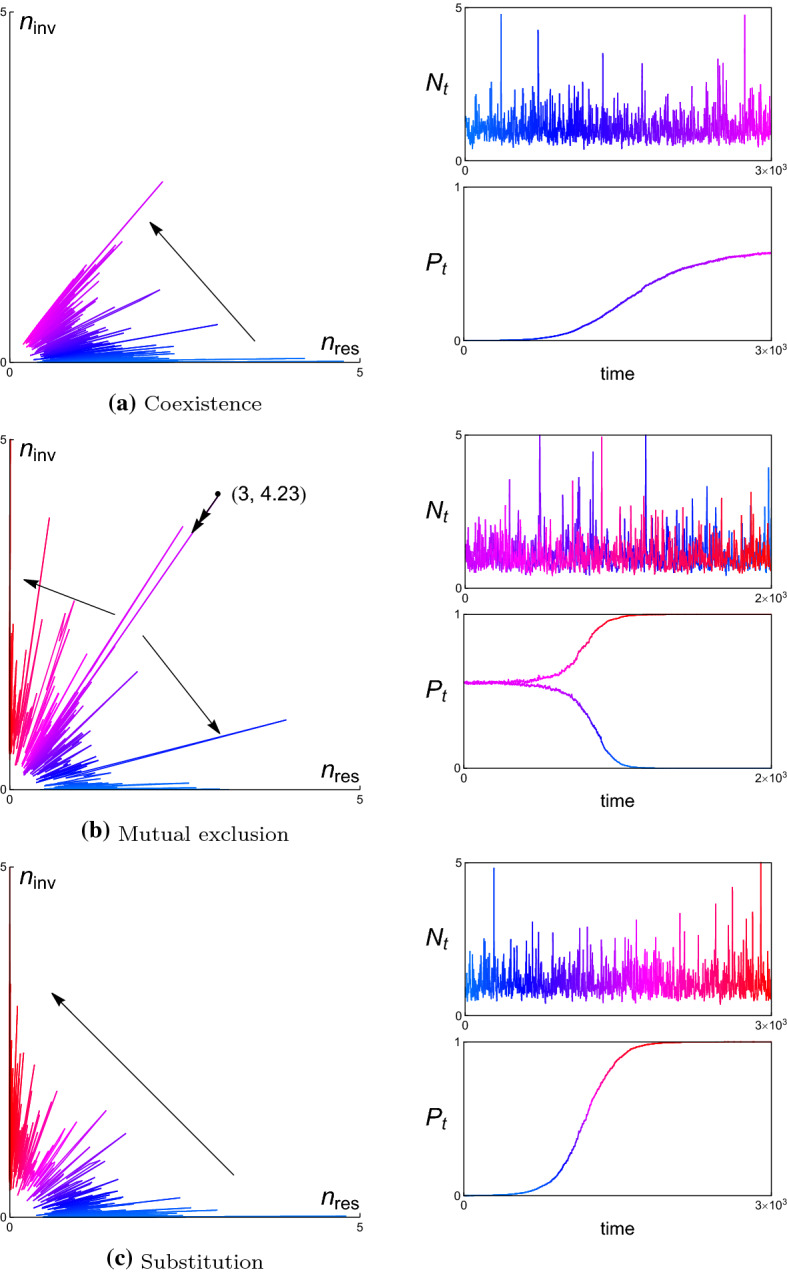
Simulated population trajectories in the phase plane and associated dynamics of the total population size $N_{t}=n_{res,t}+n_{inv,t}$ and the relative population size $P_{t}=n_{inv,t}/N_{t}$ for strategy pairs **(a)** $(x,y)=(0.48,0.51)$ with corresponding invasion fitnesses ${\mathcal {S}}_{x}(y)=0.00454545>0$ and ${\mathcal {S}}_{y}(x)=0.00346302>0$, **(b)** $(x,y)=(-0.53,-0.47)$ with corresponding invasion fitnesses ${\mathcal {S}}_{x}(y)=-\,0.0179815<0$ and ${\mathcal {S}}_{y}(x)=-\,0.0143759<0$, and **(c)** $(x,y)=(0.55,0.51)$ with corresponding invasion fitnesses ${\mathcal {S}}_{x}(y)=0.0064304>0$ and ${\mathcal {S}}_{y}(x)=-\,0.0064304<0$, where other parameter values see Fig. 5

Figure 5a shows the PIP and the MIP corresponding to (4.5) with parameters $(c_{0},c_{1},c_{2},c_{3},c_{4},c_{5})=(1,-11,11,-4,1,0.5)$. The features of these two plots are similar to that of Kisdi et al. (2001) which also studies the evolutionary of strategy *x* but for a deterministic Lotka-Volterra model with $\tilde{r}_{t}(x)=\widetilde{K}_{t}(x)=1$ for all $x\in {\mathcal {X}}$ and for all $t\ge 0$. Away from evolutionarily singular strategies $x^{*}_{er}$ (evolutionary repeller) and $x^{*}_{bp}$ (evolutionary branching point), it follows from Theorem 1 that every successful invasion of mutant *y* in a sufficiently small neighborhood of resident *x* will takeover the population. Further, directional evolution proceeding by successive invasions and substitutions with small phenotypic effect leads to an evolutionary branching point $x^{*}_{bp}$ provided that the initial strategy value is above $x^{*}_{er}$, otherwise is towards low-valued strategies if the initial strategy value is below $x^{*}_{er}$. Both $x^{*}_{er}$ and $x^{*}_{bp}$ satisfy ${\mathcal {C}}_{22}+{\mathcal {C}}_{11}\ne 0$, so that we can apply Theorem 2 to all strategies in a neighborhood of $x^{*}_{er}$ or $x^{*}_{bp}$. Having approached $x^{*}_{bp}$, the population undergoes evolutionary branching that gives rise to two district strategies coexistence. Figure 6a gives a simulated population trajectory in the phase plane for the coexistence of strategies $x=0.48$ and $y=0.51$ with corresponding invasion fitnesses ${\mathcal {S}}_{x}(y)>0$ and ${\mathcal {S}}_{y}(x)>0$. The dynamics of $P_{t}$ shows that the relative population size of the invader *y* asymptotically tends to an interior value of (0, 1).

To find the case of mutual exclusion, we choose two strategies lie in the white area of the MIP in Fig. 5a and close to the $x^{*}_{er}$. Figure 6b gives two simulated population trajectories in the phase plane for strategies $x=-\,0.53$ and $y=-\,0.47$ with corresponding invasion fitnesses ${\mathcal {S}}_{x}(y)<0$ and ${\mathcal {S}}_{y}(x)<0$, provided that initial population states are identical. In the phase plane, we see that one simulated population trajectory eventually reaches $n_{res}$-axis, while the other trajectory eventually reaches $n_{inv}$-axis. The corresponding dynamics of $P_{t}$ shows a simulated trajectory asymptotically tends to 0 but the other one asymptotically tends to 1.

Figure 5b shows the PIP and the MIP corresponding to (4.5) with parameters $(c_{0},c_{1},c_{2},c_{3},c_{4},c_{5})=(1,-2,0,-2,1,0.5)$. Now the lower singularity is still an evolutionary repeller, while the upper singularity becomes an evolutionarily stable strategy. What’s more, the associated ${\mathcal {C}}_{22}+{\mathcal {C}}_{11}=0$ but ${\mathcal {C}}_{22}\ne 0$ and ${\mathcal {C}}_{11}\ne 0$ for both two evolutionarily singular strategies $x^{*}_{er}$ and $x^{*}_{ess}$. Therefore, we apply Theorem 3 to all strategies in a neighborhood of $x^{*}_{er}$ or $x^{*}_{ess}$, in which substitution is the unique outcome of an invasion event. Figure 6c gives a simulated population trajectory in the phase plane for strategies $x=0.55$ and $y=0.51$ with corresponding invasion finesses ${\mathcal {S}}_{x}(y)>0$ and ${\mathcal {S}}_{y}(x)<0$. The dynamics of $P_{t}$ verifies that the invader *y* substitutes the resident *x* eventually.

### A structured SIRS epidemic model

Consider a structured SIRS epidemic model for the evolution of viral type. For a given moment *t* and $i=1,\dots ,k$, let $S_{t}$ be the numbers of susceptible individuals, $I_{i,t}$ be the numbers of individual infected by a virus $x_{i}\in {\mathbb {R}}_{+}:={\mathcal {X}}$, and $R_{i,t}$ be the number of recovered individual whose is infected by the virus $x_{i}$. Let $M_{t}$ be the total number of susceptible, infected and recovered individuals at time *t*, i.e.,

$$\begin{aligned} M_{t}=S_{t}+\sum _{j}I_{j,t}+\sum _{j}R_{j,t}. \end{aligned}$$

Assume that all individuals are born free of the disease at a constant rate $\varLambda >0$, and all individuals naturally die at a density-dependent rate $\delta M_{t}$ with constant $\delta >0$. In a well-mixed population, the probability of a contact being with an infected individual of $x_{i}$ is given by $\frac{I_{i,t}}{M_{t}}$. The probability of that contact giving rise to an infection of $x_{i}$ is given by ${\tilde{\beta }}_{t}(x_{i})$, which is commonly called transmission rate. Infectious individuals of $x_{i}$ recover at a rate ${\tilde{\gamma }}_{t}(x_{i})$ and disease-caused die at a rate ${\tilde{\alpha }}_{t}(x_{i})$. Recovered individuals of $x_{i}$ lose their protection against a reinfection and become susceptible again at a rate ${\tilde{\zeta }}_{t}(x_{i})$. Here ${\tilde{\beta }}_{t}(x_{i})$, ${\tilde{\gamma }}_{t}(x_{i})$, ${\tilde{\alpha }}_{t}(x_{i})$ and ${\tilde{\zeta }}_{t}(x_{i})$ are all positive, time-varying and virus-dependent. For simplicity, we assume that the transmission rate fluctuates around a virus-dependent value in the way: ${\tilde{\beta }}_{t}(x_{i})=\beta (x_{i})\exp (\rho _{1}\theta _{t})$ with $\beta (x_{i})>0$ for all $x_{i}\in {\mathcal {X}}$, where the $\{\theta _{t}\}_{t\geqslant 0}$ is a stationary Ornstein-Uhlenbeck process generated by (4.2) and $\rho _{1}$ is a scaling parameter to measure the effect of $\theta _{t}$ on the transmission rate. Likewise, ${\tilde{\gamma }}_{t}(x_{i})$, ${\tilde{\alpha }}_{t}(x_{i})$ and ${\tilde{\zeta }}_{t}(x_{i})$ fluctuate in time in the same way as ${\tilde{\beta }}_{t}(x_{i})$ but the scaling parameters are different, i.e., ${\tilde{\gamma }}_{t}(x_{i})=\gamma (x_{i})\exp (\rho _{2}\theta _{t})$, ${\tilde{\alpha }}_{t}(x_{i})=\alpha (x_{i})\exp (\rho _{3}\theta _{t})$ and ${\tilde{\zeta }}_{t}(x_{i})=\zeta (x_{i})\exp (\rho _{4}\theta _{t})$ with $\gamma (x_{i}),\alpha (x_{i})$, $\zeta (x_{i})>0$ for all $x_{i}\in {\mathcal {X}}$. Assume further that one disease-free individual is infected with a certain virus, it cannot be infected by any other virus. Under these assumptions, a structured SIRS epidemic model is given by the following differential equations:

4.6 $$\begin{aligned} \begin{aligned} {\dot{S}}_{t}&=\varLambda M_{t}-\sum _{j}{\tilde{\beta }}_{t}(x_{j})\frac{I_{j,t}}{M_{t}} S_{t}-\delta M_{t}S_{t}+\sum _{j}{\tilde{\zeta }}_{t}(x_{j})R_{j,t}, \\ \bigg (\begin{array}{c} {\dot{I}}_{i,t} \\ {\dot{R}}_{i,t} \end{array}\bigg )&=\underbrace{\bigg (\begin{array}{cc} {\tilde{\beta }}_{t}(x_{i})\tfrac{S_{t}}{M_{t}} -{\tilde{\gamma }}_{t}(x_{i})-{\tilde{\alpha }}_{t}(x_{i})-\delta M_{t} &{} 0\\ {\tilde{\gamma }}_{t}(x_{i}) &{} -\,{\tilde{\zeta }}_{t}(x_{i})-\delta M_{t} \end{array}\bigg )}_{\textstyle :=F(x_{i},S_{t},M_{t},\theta _{t})} \\&\ \ \ \times \bigg (\begin{array}{c} I_{i,t} \\ R_{i,t} \end{array}\bigg ),\ \ i=1,\dots ,k. \end{aligned} \end{aligned}$$

**Fig. 7:**
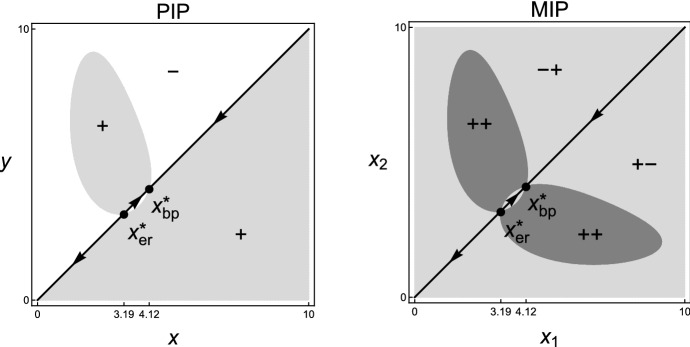
Pairwise Invasibility Plot (PIP) and corresponding Mutual Invasibility Plot (MIP) for the small-noise approximation of the invasion fitness (4.9) for $\alpha (x)=x$, $\beta (x)=5.65+\tfrac{2x^{2}}{6+0.1x^{2}}$, $\gamma (x)=0.3$ and $\zeta (x)=\exp (-0.5x^{2})$. We further have ${\mathcal {S}}_{x}(y)=\big (\beta (y)-\beta (x)\big )\Big (\frac{\widehat{S}}{\widehat{M}}+\frac{1}{2}g(\widehat{S},\widehat{M},{\hat{\theta }})\mathrm {Cov}_{(S,M,\theta )}(0)\Big )-\big (\alpha (y)-\alpha (x)\big )+{\mathcal {O}}(\rho _{1}^{2})$, where $\widehat{S}$ and $\widehat{M}$ are positive equilibrium values of the deterministic resident system, and ${\hat{\theta }}$ is the expectation of the process $\{\theta _{t}\}_{t\ge 0}$, and $g(\widehat{S},\widehat{M},{\hat{\theta }})$ is the second derivative of $\exp (\rho _{1}\theta )\frac{S}{M}$ with respect to $(S,M,\theta )$ evaluated at $(\widehat{S},\widehat{M},{\hat{\theta }})$, and $\mathrm {Cov}_{(S,M,\theta )}(0)$ is the covariance matrix in the components of $(S,M,\theta )$ at the stationary distribution of the monomorphic resident system. Notice that $\widehat{S}$, $\widehat{M}$ and $\mathrm {Cov}_{(S,M,\theta )}(0)$ are functions of the resident strategy *x*. For the interpretations of local areas, markers and arrows in the PIP and the MIP see Fig. 5. Parameter values $\varLambda =3$, $\delta =0.2$, $a=2$, $b=1$, $\rho _{1}=0.1$ and $\rho _{2}=\rho _{3}=\rho _{4}=0$

Following Sect. 3, let

$$\begin{aligned} n_{i,t}=(n^{1}_{i,t},n^{2}_{i,t})^{\top }=(I_{i,t},R_{i,t})^{\top },\ v_{i,t}=(v^{1}_{i,t},v^{2}_{i,t})^{\top }=\bigg (\frac{n^{1}_{i,t}}{\Vert n_{i,t}\Vert _{1}},\frac{n^{2}_{i,t}}{\Vert n_{i,t}\Vert _{1}}\bigg )^{\top }, \end{aligned}$$

where the state space of $v_{i,t}$ is $\varDelta =\big \{v\in [0,1]^{2}:\Vert v\Vert _{1}=1\big \}$. Then the dynamics of (4.6) are equivalent to

$$\begin{aligned} \begin{aligned} \Vert n_{i,t}\Vert _{1}^{\varvec{\cdot }}&= \mathbb {1}^{\top }F(x_{i},S_{t},M_{t},\theta _{t})v_{i,t}\Vert n_{i,t}\Vert _{1}, \\ {\dot{v}}_{i,t}&= \big (F(x_{i},S_{t},M_{t},\theta _{t})-\mathbb {1}^{\top }F(x_{i},S_{t},M_{t},\theta _{t})v_{i,t}{\mathbb {I}}\big )v_{i,t}, \\ \dot{S_{t}}&= \varLambda M_{t}-\sum _{j}{\tilde{\beta }}_{t}(x_{j})\frac{S_{t}}{M_{t}}v^{1}_{j,t}\Vert n_{j,t}\Vert _{1}-\delta M_{t}S_{t}+\sum _{j}{\tilde{\zeta }}_{t}(x_{j})v^{2}_{j,t}\Vert n_{j,t}\Vert _{1}, \\ M_{t}&= S_{t}+\sum _{j}\Vert n_{j,t}\Vert _{1}, \end{aligned} \end{aligned}$$

which can be written as the form specified in (3.2) with

$$\begin{aligned} \begin{aligned} e_{t}=(e_{1,t},e_{2,t})&= (S_{t},M_{t}),\\ G_{1}(e_{t},\theta _{t})&= (\varLambda -\delta e_{1,t})e_{2,t},\\ H_{1}(x_{j},e_{t},\theta _{t})&= \Big (-{\tilde{\beta }}_{t}(x_{j})\frac{e_{1,t}}{e_{2,t}},\ {\tilde{\zeta }}_{t}(x_{j})\Big ),\\ G_{2}(e_{t},\theta _{t})&= e_{1,t},\\ H_{2}(x_{j},e_{t},\theta _{t})&= 1. \end{aligned} \end{aligned}$$

It is easy to show that $M_{t}\le \frac{\varLambda }{\delta }$ for *t* sufficiently large. Thus, the state space of the dynamics of $Z_{t}=\big (\Vert n_{1,t}\Vert _{1},\dots ,\Vert n_{k,t}\Vert _{1},v_{1,t},\dots ,v_{k,t},e_{t},\theta _{t}\big )$ is ${\mathcal {Z}}=\big [0,\frac{\varLambda }{\delta }\big ]^{k}\times \varDelta ^{k}\times \big [0,\frac{\varLambda }{\delta }\big ]^{2}\times {\mathbb {R}}$, the extinction set ${\mathcal {Z}}_{0}=\big \{z\in {\mathcal {Z}}:\min _{i}\Vert n_{i}\Vert _{1}=0\big \}$, and the $\eta$-neighborhood of the extinction set ${\mathcal {Z}}_{\eta }=\big \{z\in {\mathcal {Z}}:\min _{i}\Vert n_{i}\Vert _{1}\le \eta \big \}$. The partial derivatives $F^{(i,j)}$, $G^{(i,j)}_{1}$, $H^{(i,j)}_{1}$, $G^{(i,j)}_{2}$ and $H^{(i,j)}_{2}$ exist and are locally Lipschitz continuous and measurable in $(e,\theta )$ for all strategies and for all nonnegative integers $i,j\le 2$.

**Fig. 8:**
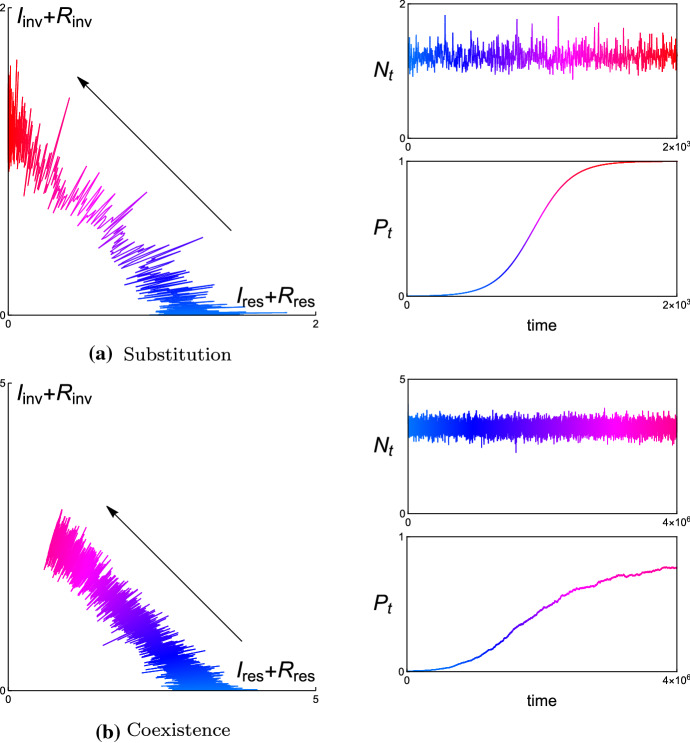
Simulated population trajectories in the phase plane and associated dynamics of the total population size $N_{t}=(I_{res,t}+R_{res,t})+(I_{inv,t}+R_{inv,t})$ and the relative population size $P_{t}=(I_{inv,t}+R_{inv,t})/N_{t}$ for strategy pairs **(a)** $(x,y)=(8.7,8.68)$ with corresponding invasion fitnesses ${\mathcal {S}}_{x}(y)\approx 0.00708633>0$ and ${\mathcal {S}}_{y}(x)\approx -0.00709391<0$, and **(b)** $(x,y)=(4.1158,4.1238)$ with corresponding invasion fitnesses ${\mathcal {S}}_{x}(y)\approx 2.27275\times 10^{-6}>0$ and ${\mathcal {S}}_{y}(x)\approx 2.27315\times 10^{-6}>0$, where parameter values refer to Fig. 7

We now start from a monomorphic population of viral type *x* with the total population size $\Vert n_{t}\Vert _{1}$ and the population structure $v_{t}$. To proceeding the further analysis, we need to know when a given viral type *x* becomes a resident, i.e., it successfully establishes in the virgin environment. The dynamics on the boundary of $\big (\Vert n_{t}\Vert _{1},S_{t},M_{t}\big )$-space globally converges to a unique stable equilibrium $\big (0,\frac{\varLambda }{\delta },\frac{\varLambda }{\delta }\big )$, provided that $S_{0}\ne 0$. Hence, the ergodic measure on the boundary of $\big (\Vert n_{t}\Vert _{1},S_{t},M_{t}\big )$-space is the Dirac measure at $\big (0,\frac{\varLambda }{\delta },\frac{\varLambda }{\delta }\big )$. Substitution of this stable equilibrium into the equation $v_{t}$ gives

4.7 $$\begin{aligned} {\dot{v}}^{1}_{t}= & {} \underbrace{\Big ({\tilde{\beta }}_{t}(x)-{\tilde{\gamma }}_{t}(x)-{\tilde{\alpha }}_{t}(x)-\varLambda -\big ({\tilde{\beta }}_{t}(x)-{\tilde{\alpha }}_{t}(x)-\varLambda \big )v^{1}_{t}+\big ({\tilde{\zeta }}_{t}(x)+\varLambda \big )v^{2}_{t}\Big )}_{\textstyle :=h(v^{1}_{t},v^{2}_{t},\theta _{t})}v^{1}_{t}\nonumber \\ v^{2}_{t}= & {} 1-v^{1}_{t}. \end{aligned}$$

Providing that the expectation

4.8 $$\begin{aligned} {\mathbb {E}}\big [{\tilde{\beta }}(x)-{\tilde{\gamma }}(x)-{\tilde{\alpha }}(x)-\varLambda \big ]>0, \end{aligned}$$

the dynamics of (4.7) consist of a Dirac measure at the unstable equilibrium $v=(0,1)$ and invariant probability measures $\hat{v}$ supported on $\varDelta \setminus \{(0,1)\}$. Let $\mu$ be the product of the Dirac measure at $\big (\Vert n\Vert _{1},S,M\big )=\big (0,\frac{\varLambda }{\delta },\frac{\varLambda }{\delta }\big )$ and the $\hat{v}$. Then $\lambda _{x}(\mu )={\mathbb {E}}\big [\big ({\tilde{\beta }}(x)\frac{S}{M}-{\tilde{\alpha }}(x)-\delta M\big )v^{1}-\big ({\tilde{\zeta }}(x)+\delta M\big )v^{2}\big ]$ corresponds to the per-capita growth rate of $\Vert n_{t}\Vert _{1}$ with respect to $\mu$. From the dynamics of $v^{1}_{t}$ we have that with probability one $\lim _{t\rightarrow +\infty }\frac{1}{t}\log v^{1}_{t}=\lim _{t\rightarrow +\infty }\frac{1}{t}\int _{0}^{t}h(v^{1}_{s},v^{2}_{s},\theta _{s})ds={\mathbb {E}}\big [h(v^{1},v^{2},\theta )\big ]=0$ for $(v^{1}_{0},v^{2}_{0})\in \varDelta \setminus \{(0,1)\}$, which implies that $\lambda _{x}(\mu )={\mathbb {E}}\big [{\tilde{\beta }}(x)-{\tilde{\gamma }}(x)-{\tilde{\alpha }}(x)-\varLambda \big ]$. Thus, if (4.8) holds, a similar argument as the first example implies that the population of viral type *x* is non-growing on the long-run and stochastically persistent.

Once virus *x* becomes a resident, the invasion fitness of an initially rare mutant *y* in resident *x* is given by

4.9 $$\begin{aligned} {\mathcal {S}}_{x}(y)={\mathbb {E}}\big [\big ({\tilde{\beta }}(y)-{\tilde{\beta }}(x)\big )\tfrac{S}{M}-\big ({\tilde{\gamma }}(y)-{\tilde{\gamma }}(x)\big )-\big ({\tilde{\alpha }}(y)-{\tilde{\alpha }}(x)\big )\big ]. \end{aligned}$$

Here we have used the principle of selective neutrality of residents (i.e., ${\mathcal {S}}_{x}(x)=0$ for all $x\in {\mathcal {X}}$) to derive (4.9). Generally, there isn’t an explicit expression of (4.9) in terms of strategies *x* and *y*. Thus the numerical PIP and MIP are based on a small-noise approximation of (4.9) (see e.g., Vilar and Rubi 2018).

Figure 7 shows the PIP and the MIP corresponding to a small-noise approximation of (4.9) (ref to the legend). Away from the two evolutionarily singular strategies $x^{*}_{er}$ and $x^{*}_{bp}$, every successful invasion of mutant *y* in a sufficiently small neighborhood of resident *x* will takeover the population. Figure 8a gives a simulated population trajectory in the phase plane for strategies $x=8.7$ and $y=8.68$ with corresponding invasion fitnesses ${\mathcal {S}}_{x}(y)>0$ and ${\mathcal {S}}_{y}(x)<0$. The dynamics of $P_{t}$ shows that the invader *y* will eventually oust the resident *x* and become the new resident. Close to the two evolutionarily singular strategies, Figure 8b gives a simulated population trajectory in the phase plane for strategies $x=4.1158$ and $y=4.1238$ with corresponding invasion fitnesses ${\mathcal {S}}_{x}(y)>0$ and ${\mathcal {S}}_{y}(x)>0$. The dynamics of $P_{t}$ shows that the relative population size asymptotically tends to an interior value of (0, 1), which implies that the two viral types can coexist.

### A prey-predator model: continuation of Example 1

In order to show that the deterministic model of Example 1 belongs to the general class of models considered in this paper, let

$$\begin{aligned} \begin{aligned} G_{1}(e_{t})&= -\,\delta e_{1,t}, \\ H_{1}(x_{j},e_{t})&= \frac{\gamma \big (1+\beta he_{2,t}\big )\frac{\beta e_{1,t}}{(1+\beta he_{2,t})(1+x_{j}e_{1,t})}}{T\big (\sigma (1+\beta h e_{2,t})+(1-\lambda )\alpha e_{1,t}\big )}, \\ G_{2}(e_{t})&=0, \\ H_{2}(x_{j},e_{t})&= \frac{1}{1+x_{j}e_{1,t}}, \end{aligned} \end{aligned}$$

then the deterministic model of Example 1 can be written as the general form (2.1). The partial derivatives $f^{(i,j)}$, $G^{(i,j)}_{1}$, $H^{(i,j)}_{1}$, $G^{(i,j)}_{2}$ and $H^{(i,j)}_{2}$ exist for all strategies and for all nonnegative integers $i,j\le 2$, which satisfy Assumption **A2**.

**Fig. 9:**
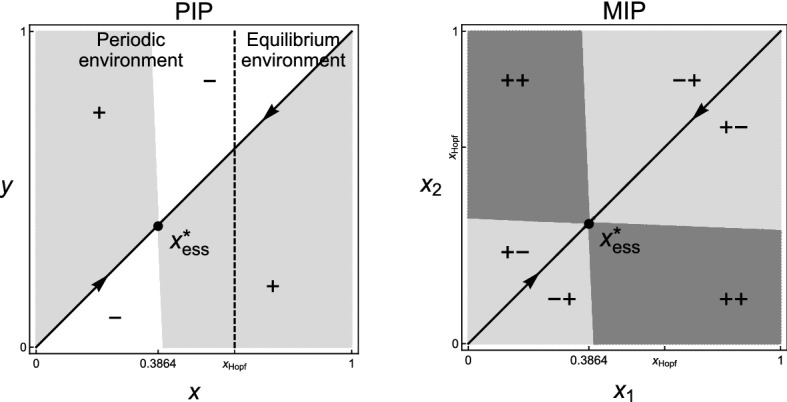
Pairwise Invasibility Plot (PIP) and corresponding Mutual Invasibility Plot (MIP) of the invasion fitness (4.10). For the interpretations of local areas, markers and arrows in the PIP and the MIP see Fig. 5. Parameter values $a=c=2$, $\mu =\delta =h=T=1$, $\gamma =3$, $\lambda =0.6$, $\sigma =0.7$, $\alpha =0$, and $\beta =6$. Further, the Hopf bifurcation point $x_{\text {Hopf}}=0.6289$

The evolutionary dynamics of the deterministic model of Example 1 has been well studied by Lehtinen and Geritz (2019). We are interesting in the evolution of timidity of the prey with the non-equilibrium resident dynamics. When only a single prey type *x* is present, the system has a stable interior equilibrium at $x=1$. Decreasing *x* destabilises the equilibrium through a supercritical Hopf bifurcation at $x_{\text {Hopf}}=0.6289$, after which periodic attractors are present (ref to Lehtinen and Geritz (2019, Fig. 3B)). In a periodic resident environment set by a single resident type *x*, the invasion fitness of an initially rare mutant *y* is given by

4.10 $$\begin{aligned} {\mathcal {S}}_{x}(y)=\frac{1}{\tau (x)}\int ^{\tau (x)}_{0}f(y,e_{t})dt, \end{aligned}$$

where $\tau (x)$ is the period (ref to Metz et al. 1992).

**Fig. 10:**
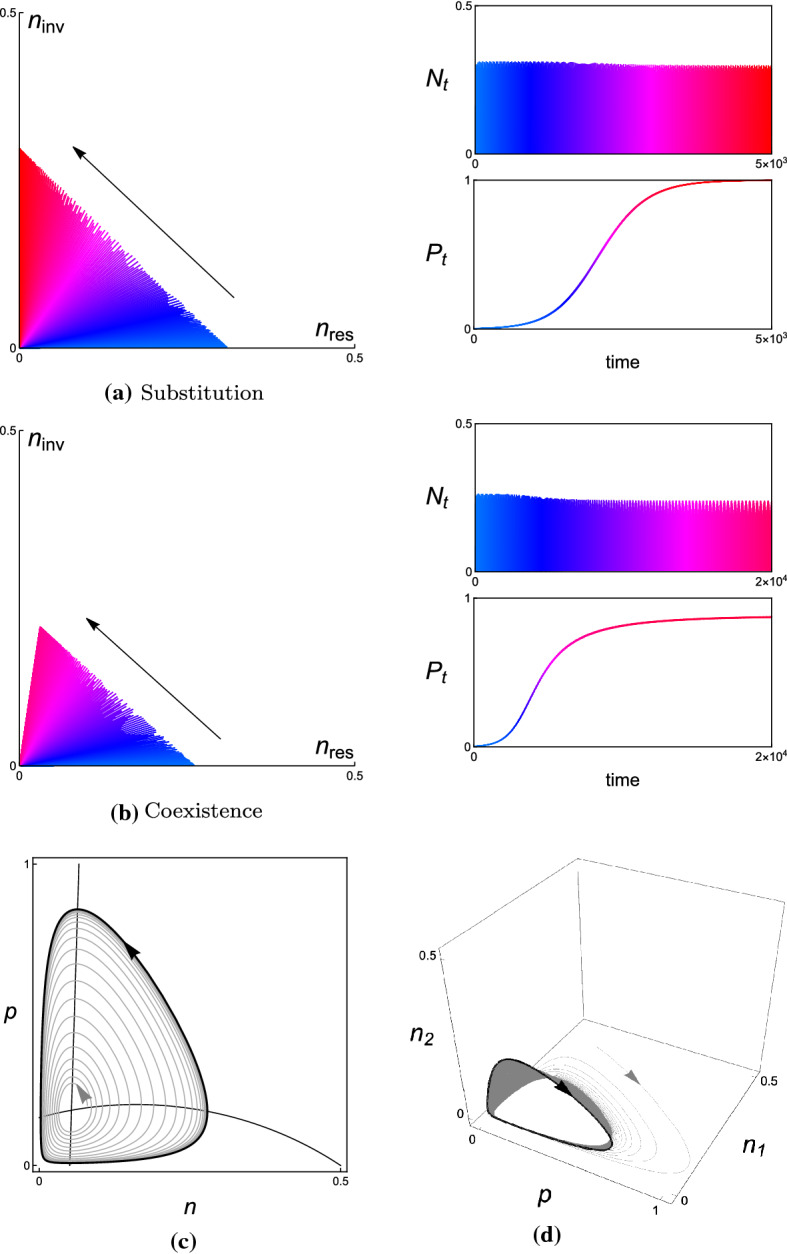
Simulated population trajectories in the phase plane and associated dynamics of the total population size $N_{t}=n_{res,t}+n_{inv,t}$ and the relative population size $P_{t}=n_{inv,t}/N_{t}$ for strategy pairs **(a)** $(x,y)=(0.27,0.3)$ with corresponding invasion fitnesses ${\mathcal {S}}_{x}(y)\approx 0.002141>0$ and ${\mathcal {S}}_{y}(x)\approx -0.001671<0$, and **(b)** $(x,y)=(0.3764,0.4314)$ with corresponding invasion fitnesses ${\mathcal {S}}_{x}(y)\approx 0.000277>0$ and ${\mathcal {S}}_{y}(x)\approx 0.001496>0$. **(c)** the periodic attractor of the monomorphic model with resident strategy $x=0.3$. **(d)** the periodic attractor of the dimorphic model with coexistence strategies $(x_{1},x_{2})=(0.3764,0.4314)$. Parameter values are the same as Fig. 9

Figure 9 shows the PIP and the MIP corresponding to (4.10) with the same parameters as Lehtinen and Geritz (2019, Fig. 4), in which the monomorphic resident environment is an equilibrium environment if $x\ge x_{\text {Hopf}}$ and becomes a periodic environment if $x<x_{\text {Hopf}}$. In the periodically resident environment, away from the evolutionarily singular strategy $x^{*}_{ess}$, it follows from Theorem 1 that successful invasion of mutant *y* in resident *x* will takeover the population. Figure 10a gives a simulated population trajectory in the phase plane for strategies $x=0.27$ and $y=0.3$ with corresponding invasion fitnesses ${\mathcal {S}}_{x}(y)>0$ and ${\mathcal {S}}_{y}(x)<0$. The total population size of $N_{t}=n_{res,t}+n_{inv,t}$ varies significantly in time. However, the relative population size $P_{t}=\frac{n_{inv,t}}{N_{t}}$ changes slowly in time and asymptotically increases from 0 to 1. The dynamics of $P_{t}$ implies that the invader *y* will oust the resident *x* and becomes a new resident. Figure 10c shows the periodic attractor of the monomorphic model with the new resident strategy $x=0.3$. In the neighborhood of $x^{*}_{ess}$, we can apply Theorem 2 to predict the population dynamical outcomes of an invasion event. If both strategies of the resident and the mutant are in the gray area of MIP, it follows from Theorem 2 that the resident and the mutant can coexist eventually (notice that all coexistence strategies are not evolutionarily stable). Figure 10b gives a simulated population trajectory in the phase plane for the coexistence strategies $(x,y)=(0.3764,0.4314)$ with corresponding invasion fitnesses ${\mathcal {S}}_{x}(y)>0$ and ${\mathcal {S}}_{y}(x)>0$. The total population size $N_{t}$ still fluctuates significantly in time. but the relative population size $P_{t}$ slowly and asymptotically tends to an interior value of (0, 1). The dynamics of $P_{t}$ verifies that the invader *y* eventually coexists with the resident *x*. Figure 10d shows the periodic attractor of the dimorphic model with coexistence strategies $(x_{1},x_{2})=(0.3764,0.4314)$.

## Discussion

Our main result is the complete classification of generic population dynamical outcomes of resident-invader dynamics in fluctuating environments when the invader and the resident have similar strategies. The outcomes are essentially “Lotka-Volterra”: (i) one strategy ousts the other if the one can invade a population of the other but not the other way around, i.e., invasion implies substitution; (ii) the two strategies coexist if they can mutual invade; (iii) they mutually exclude one another if neither can invade a population of the other. Which of the four Lotka-Volterra-type outcomes occurs depends only on the signs of the invasion fitnesses. In a one-dimensional strategy space or a one-dimensional parameterization of a multi-dimensional strategy space (see e.g., Kisdi 2015), invasion implies substitution away from an evolutionarily singular strategy, while all four Lotka-Volterra-type outcomes are possible close to an evolutionarily singular strategy. In a multi-dimensional strategy space, however, the situation is generally complicated because all four Lotka-Volterra-type outcomes may occur also away from an evolutionarily singular strategy.

We extend and generalize previous results of resident-invader dynamics of similar strategies to models incorporating (i) explicit feedback environments with their own dynamics, (ii) scalar-valued as well as vector-valued strategies, (iii) unstructured as well as structured populations, (iv) monomorphic as well as polymorphic resident populations, and (v) non-equilibrium resident population dynamics as well as resident dynamics with stochastic (or deterministic) drivers. Although we show all results for models with a monomorphic resident population (i.e., single resident phenotype), the generalization of them to polymorphic resident populations (i.e., multiple resident phenotypes) is straight forward because of the way we modelled the environment feedback loop. Arbitrarily polymorphic resident populations can be accounted for by treating the corresponding population sizes of the extra resident phenotypes as environmental feedback variables. In the next paragraphs we focus on the differences between our work and previous studies.

For a class of unstructured population models with point equilibria, Geritz (2005) has shown that the resident-invader dynamics generically behaves like the Lotka-Volterra competition model by using of the Poincaré-Bendixson theorem. The results hold for vector-valued strategies but were proved only for a feedback environment that is given as an explicit linear function of the resident and invader population sizes. However, environmental feedback variables such as resources, predators, or competitors are often given implicitly (e.g., by differential equations) as opposed to explicitly. Our implicit representation is more general than the explicit and liner environmental feedback used in Geritz (2005).

Dercole and Geritz (2016) has the same aims and results as Geritz (2005), but it is more general in some aspects but less general in others, and it uses a basically different mathematical approach, i.e., time-scale arguments. The results only apply to unstructured population models with point equilibria and scalar-valued strategies, but the formulation of environmental feedback is very general—probably as general as in our approach (in their formulation, they allow the resident and the invader to interact with finitely many other populations whose corresponding sizes are packed in the feedback variable $e_{t}$. The dynamics of $e_{t}$ is govern by a differential equation, in which the growth rate is an implicit function of the resident, the invader, and the environmental variable itself. Given four structural properties of the growth rate of the $e_{t}$ as well as the per-capita growth rates of the resident and the invader, their formulation generalizes the law of mass action (see Dercole 2016). The generalization takes into account that pairwise interactions can depend on the concomitant activities of the encountered individuals, which leads a nonlinear density dependence in the per-capita growth rates of the resident and the invader). The paper also studies some mathematically degenerate cases which confirm the results of Priklopil (2012) on unprotected coexistence of two strategies near an evolutionarily singular strategy.

The “invasion implies substitution” outcome for unstructured population models with point equilibria also can be found in Dercole and Rinaldi (2008, Section 3.4) and Oba and Kigami (2018).

Our paper is the first to study resident-invader dynamics in fluctuating environments. We study the resident-invader dynamics as a stochastic fast-slow system where the total population size of the resident and the invader is the fast variable and the relative population size of the invader is the slow variable. We show that trajectories of the slow variable on slow timescale are well approximated by that of an associated averaged system, and the stability of the averaged system depends on invasion criteria alone. From these results, Theorems 1, 2 and 3 give the complete classification of generic population dynamical outcomes of an invasion event.

For a class of structured population models with point equilibria, recently, Priklopil and Lehmann (2019) have shown that invasion implies substitution for ecological communities with finite-class-structured populations of scalar-valued strategies. Their approach is based on the analysis of a weighted average of the relative invader population sizes in each class, where the weighting coefficient is the class-specific reproductive value (see also Lion 2018a). However, it is not clear how their method can be used to give a full classification of the generic population dynamical outcomes of an invasion event. Moreover, it is not clear how their approach can be applied to fluctuating environments.

Cantrell et al. (2017) has extended the *tube theorem* of Geritz et al. (2002) and the *invasion implies fixation theorem* of Geritz (2005) to a class of reaction-diffusion models for understanding evolution of dispersal in space. Their focus is on a dimorphic system (i.e., one resident and one invader) of infinite-dimensional structured populations of scalar-valued strategies.

When populations are in a fluctuating environment, we illustrate how to generalize the results of unstructured population models to a class of structured population models, but only for finite-class-structured populations. The extension of our results to populations with a more general structure (e.g., size distributions, continuous age and spatial diffusion) we leave for future work.

As mentioned in the Introduction, there are serval different (not necessarily equivalent) definitions of stochastic persistence for a resident population. Most of them can be summed up from the “ensemble” perspective in terms of transition probabilities (see e.g., Chesson 1982; Chesson and Ellner 1989; Li and Mao 2009) or the “typical trajectory” perspective in terms of empirical measures (i.e., how typical sample trajectories of the population process are distributed in time, see e.g., Benaïm and Schreiber 2009, 2019; Schreiber et al. 2011; Roth and Schreiber 2014; Benaïm 2018) for the long-term population dynamics. Different definitions often give different interpretations of the population dynamics. Extending our results to models with different kinds of resident dynamics would be useful for the applicability of the theory of adaptive dynamics. Our definition of resident persistence follows Schreiber et al. (2011) and Benaïm (2018) is a rather weak one. This means that our results also apply to more restrictive definitions.

## Appendix A Rewrite the environmental variable of Example 2 as the general form (2.1b)

Still denote the environmental variable by $e_{t}$. Let ${\mathcal {E}}$ be the set of all functions $e:{\mathcal {X}}\mapsto {\mathbb {R}}^{2}_{+}$ of the form

$$\begin{aligned} \begin{aligned} e_{t}(\cdot )&= (e_{1,t},e_{2,t})(\cdot ) \\&= \big (e_{1,t},(e_{21,t},e_{22,t},e_{23,t},e_{24,t})\big )(\cdot ) \\&= \Bigg (0,\ \bigg (\sum _{j}a(\cdot ,x_{j})n_{j,t},\ \sum _{j}h(\cdot )a(\cdot ,x_{j})n_{j,t}, \\&\qquad \qquad \sum _{j}\frac{a(x_{j},\cdot )}{1+h_{0}(x_{j})a_{0}(x_{j})n_{0}+e_{22,t}(x_{j})}n_{j,t},\ \sum _{j}cn_{j,t}\bigg )\Bigg ) \end{aligned} \end{aligned}$$

for $n_{j,t}\ge 0$ and $x_{j}\in {\mathcal {X}}$. Then we can define the per-capita environmental impact $H_{2}:{\mathcal {X}}\times {\mathcal {E}}\mapsto {\mathbb {R}}$ by

$$\begin{aligned} H_{2}(x_{j},e_{t})(\cdot )=\bigg (a(\cdot ,x_{j}),\ h(\cdot )a(\cdot ,x_{j}),\ \frac{a(x_{j},\cdot )}{1+h_{0}(x_{j})a_{0}(x_{j})n_{0}+e_{22,t}(x_{j})},\ c\bigg ). \end{aligned}$$

Since the strategy of sub-population *i* has infinitely many choices, $e_{2,t}$ has infinite dimensions. In addition, define the map $G_{2}:{\mathcal {E}}\times {\mathbb {R}}\mapsto {\mathbb {R}}$ by

$$\begin{aligned} G_{2}(e_{t},\theta )=(0,0,0,0). \end{aligned}$$

Hence, $e_{t}$ gets the general form (2.1b).

## Appendix B Approximation on *t*-timescale when $\epsilon \rightarrow 0$

Writing (2.5) into the following form:

$$\begin{aligned} \dot{\widetilde{Z}^{\epsilon }_{t}}=\varPhi (x+\epsilon \xi _{1},x+\epsilon \xi _{2},\widetilde{Z}^{\epsilon }_{t}), \end{aligned}$$

where

$$\begin{aligned} \widetilde{Z}^{\epsilon }_{t}=(N^{\epsilon }_{t},P^{\epsilon }_{t},e^{\epsilon }_{t},\theta _{t})\in {\mathcal {N}}\times [0,1]\times {\mathcal {E}}\times \varTheta :=\widetilde{{\mathcal {Z}}}. \end{aligned}$$

For $\epsilon =0$, we have $\widetilde{Z}^{0}_{t}=(N^{0}_{t},P^{0}_{t},e^{0}_{t},\theta _{t})\in \widetilde{{\mathcal {Z}}}$. Then (2.6) can be written as the same form, i.e.,

$$\begin{aligned} \dot{\widetilde{Z}^{0}_{t}}=\varPhi (x,x,\widetilde{Z}^{0}_{t}). \end{aligned}$$

From Assumption **A1**, $\{\widetilde{Z}^{\epsilon }_{t}\}_{t\ge 0}$ is a Markov process for any given $\epsilon$ (ref to Arnold and Kliemann (1983, Lemma 2.1)). Let $\{\widetilde{{\mathcal {T}}}^{\epsilon }_{t}\}_{t\ge 0}$ and $\{\widetilde{{\mathcal {T}}}^{0}_{t}\}_{t\ge 0}$ are the associated semigroup for Markov process $\{\widetilde{Z}^{\epsilon }_{t}\}_{t\ge 0}$ with $\epsilon >0$ and for Markov process $\{\widetilde{Z}^{0}_{t}\}_{t\ge 0}$ when $\epsilon =0$, respectively. In addition, the function $\varPhi$ requires to satisfy the following assumption:

**A3**: The partial derivatives $\varPhi ^{(i,j,k)}$ exit and are locally Lipschitz continuous and measurable in *z* for all strategies and all $\epsilon$ and for all $i,j,k\in \{0,1\}$.

For positive but small $\epsilon$, $\widetilde{Z}^{\epsilon }_{t}$ is sufficiently closed to $\widetilde{Z}^{0}_{t}$ in terms of conditional expectations as the following lemma shows.

### Lemma 3

Let $\widetilde{Z}^{\epsilon }_{0}=\widetilde{Z}^{0}_{0}=z$. Then, for all bounded and measurable function $h:\mathcal {\widetilde{Z}}\mapsto {\mathbb {R}}$, we have

$$\begin{aligned} \lim _{\epsilon \rightarrow 0}\widetilde{{\mathcal {T}}}^{\epsilon }_{t}h(z)=\widetilde{{\mathcal {T}}}^{0}_{t}h(z) \end{aligned}$$

for all $t\ge 0$, and is uniformly in $z\in \mathcal {\widetilde{Z}}$.

### Proof

For all $$t\ge 0$$

$$\begin{aligned} \widetilde{Z}^{\epsilon }_{t}-\widetilde{Z}^{0}_{t}= & {} \int _{0}^{t}\big (\varPhi (x+\epsilon \xi _{1},x+\epsilon \xi _{2},\widetilde{Z}^{\epsilon }_{s})-\varPhi (x+\epsilon \xi _{1},x+\epsilon \xi _{2},\widetilde{Z}^{0}_{s})\big )ds \\&+\,\int _{0}^{t}\big (\varPhi (x+\epsilon \xi _{1},x+\epsilon \xi _{2},\widetilde{Z}^{0}_{s})-\varPhi (x+\epsilon \xi _{1},x,\widetilde{Z}^{0}_{s})\big )ds \\&+\,\int _{0}^{t}\big (\varPhi (x+\epsilon \xi _{1},x,\widetilde{Z}^{0}_{s})-\varPhi (x,x,\widetilde{Z}^{0}_{s})\big )ds. \end{aligned}$$

Let

$$\begin{aligned} \varphi _{z}(t)={\mathbb {E}}_{z}\big [\Vert \widetilde{Z}^{\epsilon }_{t}-\widetilde{Z}^{0}_{t}\Vert ^{2}\big ]. \end{aligned}$$

Using the Cauchy–Schwartz inequality we have

$$\begin{aligned} \varphi _{z}(t)\le & {} 3{\mathbb {E}}_{z}\bigg [\bigg \Vert \int _{0}^{t}\big (\varPhi (x+\epsilon \xi _{1},x+\epsilon \xi _{2},\widetilde{Z}^{\epsilon }_{s})-\varPhi (x+\epsilon \xi _{1},x+\epsilon \xi _{2},\widetilde{Z}^{0}_{s})\big )ds\bigg \Vert ^{2} \\&\ \ \ \ \ \ +\bigg \Vert \int _{0}^{t}\big (\varPhi (x+\epsilon \xi _{1},x+\epsilon \xi _{2},\widetilde{Z}^{0}_{s})-\varPhi (x+\epsilon \xi _{1},x,\widetilde{Z}^{0}_{s})\big )ds\bigg \Vert ^{2} \\&\ \ \ \ \ \ +\bigg \Vert \int _{0}^{t}\big (\varPhi (x+\epsilon \xi _{1},x,\widetilde{Z}^{0}_{s})-\varPhi (x,x,\widetilde{Z}^{0}_{s})\big )ds\bigg \Vert ^{2}\bigg ]\\\le & {} 3t\int _{0}^{t}{\mathbb {E}}_{z}\Big [\big \Vert \varPhi (x+\epsilon \xi _{1},x+\epsilon \xi _{2},\widetilde{Z}^{\epsilon }_{s})-\varPhi (x+\epsilon \xi _{1},x+\epsilon \xi _{2},\widetilde{Z}^{0}_{s})\big \Vert ^{2}\Big ]ds \\&+\,3t\int _{0}^{t}{\mathbb {E}}_{z}\Big [\big \Vert \varPhi (x+\epsilon \xi _{1},x+\epsilon \xi _{2},\widetilde{Z}^{0}_{s})-\varPhi (x+\epsilon \xi _{1},x,\widetilde{Z}^{0}_{s})\big \Vert ^{2}\Big ]ds \\&+\,3t\int _{0}^{t}{\mathbb {E}}_{z}\Big [\big \Vert \varPhi (x+\epsilon \xi _{1},x,\widetilde{Z}^{0}_{s})-\varPhi (x,x,\widetilde{Z}^{0}_{s})\big \Vert ^{2}\Big ]ds. \end{aligned}$$

By Assumption **A3**, there exist some positive constants $K_{0}$, $K_{1}$ and $K_{2}$ such that

$$\begin{aligned} \varphi _{z}(t)\le 3tK_{0}\int _{0}^{t}\varphi _{z}(s)ds+3t^{2}(K_{1}\xi _{1}+K_{2}\xi _{2})\epsilon ^{2}. \end{aligned}$$

Then, by the Gronwall’s lemma, it follows that for all $t\in [0,T]$

$$\begin{aligned} \varphi _{z}(t)\le \epsilon ^{2}\widetilde{K}_{1}\exp (t\widetilde{K}_{0}), \end{aligned}$$

where $$\widetilde{K}_{0}=3TK_{0}$$ and $$\widetilde{K}_{1}=3T^{2}(K_{1}\xi _{1}+K_{2}\xi _{2})$$.

Let $h:\mathcal {\widetilde{Z}}\mapsto {\mathbb {R}}$ is a bounded and measurable function. By uniform continuity, there exists $\delta >0$ such that for all $z_{1},z_{2}\in \mathcal {\widetilde{Z}}$ with $\Vert z_{1}-z_{2}\Vert \le \delta$, we have $|h(z_{1})-h(z_{2})|\le \epsilon$. Using the Markov’s inequality, we obtain that for $t\in [0,T]$

$$\begin{aligned} \Big |\widetilde{{\mathcal {T}}}^{\epsilon }_{t}h(z)-\widetilde{{\mathcal {T}}}^{0}_{t}h(z)\Big |= & {} \Big |{\mathbb {E}}_{z}\big [h(\widetilde{Z}^{\epsilon }_{t})\big ]-{\mathbb {E}}_{z}\big [h(\widetilde{Z}^{0}_{t})\big ]\Big | \\\le & {} 2\Vert h\Vert {\mathbb {P}}_{z}\big \{\Vert \widetilde{Z}^{\epsilon }_{t}-\widetilde{Z}^{0}_{t}\Vert \ge \delta \big \}+\epsilon \\\le & {} 2\Vert h\Vert \delta ^{-1}{\mathbb {E}}_{z}\big [\Vert \widetilde{Z}^{\epsilon }_{t}-\widetilde{Z}^{0}_{t}\Vert \big ]+\epsilon \\\le & {} 2\Vert h\Vert \delta ^{-1}\sqrt{\varphi _{z}(t)}+\epsilon \\\le & {} \epsilon \Big (2|h|\delta ^{-1}\sqrt{\widetilde{K}_{1}\exp (t\widetilde{K}_{0})}+1\Big ). \end{aligned}$$

This inequality holds for any $T>0$, we thus get the desired result for all $t\ge 0$.

The proof is complete. $\square$

## Appendix C Proof of Lemma 1

From now on, we fix initial values $P^{\epsilon }_{0}=\bar{P}_{0}=p$ and $Z_{0}=z$. The proof of Lemma 1 will be separated into the following three steps.

Firstly, we show that the trajectory of $\bar{P}_{t_{1}}$ is sufficiently close to that of $P^{\epsilon }_{t_{1}}$ for $\epsilon >0$ small in every finite time interval.

### Lemma 4

Assume that for a small $\sigma >0$, $\int _{{\mathcal {Z}}\setminus {\mathcal {Z}}_{0}}|f(y,e,\theta )|^{2}\mu (de,d\theta )<+\infty$ for all $y\in \big \{\tilde{x}\in {\mathcal {X}}:\Vert \tilde{x}-x\Vert \le \sigma \big \}$. Then for every $T>0$, the solutions of (2.7) and (2.9) satisfy

c.1 $$\begin{aligned} {\mathbb {P}}_{(p,z)}\bigg \{\lim _{\epsilon \rightarrow 0}\sup _{0\le t_{1}\le T}| P^{\epsilon }_{t_{1}}-\bar{P}_{t_{1}}|=0\bigg \}=1 \end{aligned}$$

for all $p\in [0,1]$ and for $\mu$-almost every $z\in {\mathcal {Z}}\setminus {\mathcal {Z}}_{0}$.

### Proof

Denote

$$\begin{aligned} h(z)=f^{(1,0)}(x,e,\theta )^{\top }(\xi _{2}-\xi _{1}) \end{aligned}$$

where $z=(n,e,\theta )$. Let $\bar{h}$ be the expectation of *h*(*z*) with respect to $\mu$, i.e.,

$$\begin{aligned} \bar{h}=\int _{{\mathcal {Z}}\setminus {\mathcal {Z}}_{0}}h(z)\mu (dz)=\partial _{y}{\mathcal {S}}_{x}(y)|_{y=x}(\xi _{2}-\xi _{1}). \end{aligned}$$

For all $t_{1}\ge 0$, ignoring the term ${\mathcal {O}}(\epsilon )$ in (2.7),

$$\begin{aligned} P^{\epsilon }_{t_{1}}-\bar{P}_{t_{1}}= & {} \int _{0}^{t_{1}}\big (P^{\epsilon }_{s}(1-P^{\epsilon }_{s})h(Z_{s/\epsilon })-\bar{P}_{s}(1-\bar{P}_{s})\bar{h}\big )ds \\= & {} \int _{0}^{t_{1}}\big (P^{\epsilon }_{s}(1-P^{\epsilon }_{s})h(Z_{s/\epsilon })-\bar{P}_{s}(1-\bar{P}_{s})h(Z_{s/\epsilon })\big )ds \\&+\int _{0}^{t_{1}}\big (\bar{P}_{s}(1-\bar{P}_{s})h(Z_{s/\epsilon })-\bar{P}_{s}(1-\bar{P}_{s})\bar{h}\big )ds. \end{aligned}$$

Fixed $T>0$, using the Cauchy–Schwartz inequality we have

$$\begin{aligned} \sup _{0\le t_{1}\le T}|P^{\epsilon }_{t_{1}}-\bar{P}_{t_{1}}|^{2}\le & {} \sup _{0\le t_{1}\le T}2\bigg |\int _{0}^{t_{1}}(P^{\epsilon }_{s}-\bar{P}_{s})(1-P^{\epsilon }_{s}-\bar{P}_{s})h(Z_{s/\epsilon })ds\bigg |^{2} \\&+\sup _{0\le t_{1}\le T}2\bigg |\int _{0}^{t_{1}}\bar{P}_{s}(1-\bar{P}_{s})\big (h(Z_{s/\epsilon })-\bar{h}\big )ds\bigg |^{2} \\\le & {} \sup _{0\le t_{1}\le T}2\int _{0}^{t_{1}}|P^{\epsilon }_{s}-\bar{P}_{s}|^{2}ds\int _{0}^{T}\big |h(Z_{s/\epsilon })\big |^{2}ds \\&+\sup _{0\le t_{1}\le T}2\bigg |\int _{0}^{t_{1}}\bar{P}_{s}(1-\bar{P}_{s})\big (h(Z_{s/\epsilon })-\bar{h}\big )ds\bigg |^{2}. \end{aligned}$$

Denote

$$\begin{aligned} \alpha _{\epsilon }= & {} 2\int _{0}^{T}\big |h(Z_{s/\epsilon })\big |^{2}ds=2T\frac{1}{T/\epsilon }\int _{0}^{T/\epsilon }\big |h(Z_{s})\big |^{2}ds, \\ \beta _{\epsilon }= & {} \sup _{0\le t_{1}\le T}2\bigg |\int _{0}^{t_{1}}\bar{P}_{s}(1-\bar{P}_{s})\big (h(Z_{s/\epsilon })-\bar{h}\big )ds\bigg |^{2}. \end{aligned}$$

By the Gronwall’s lemma, it follows that

$$\begin{aligned} \sup _{0\le t_{1}\le T}|P^{\epsilon }_{t_{1}}-\bar{P}_{t_{1}}|^{2}\le \beta _{\epsilon }\exp (\alpha _{\epsilon }). \end{aligned}$$

We now focus on the limit of $\alpha _{\epsilon }$ and $\beta _{\epsilon }$ as $\epsilon \rightarrow 0$, respectively. By Birkhoff’s ergodic theorem, we obtain that for all $p\in [0,1]$ and for $\mu$-almost every $z\in {\mathcal {Z}}\setminus {\mathcal {Z}}_{0}$

c.2 $$\begin{aligned} {\mathbb {P}}_{(p,z)}\bigg \{\lim _{\epsilon \rightarrow 0}\alpha _{\epsilon }=2T\int _{{\mathcal {Z}}\setminus {\mathcal {Z}}_{0}}\big |h(z)\big |^{2}\mu (dz)<+\infty \bigg \}=1. \end{aligned}$$

Here, we have used that $\int _{{\mathcal {Z}}\setminus {\mathcal {Z}}_{0}}|f^{(1,0)}(x,e,\theta )^{\top }(\xi _{2}-\xi _{1})|^{2}\mu (de,d\theta )<+\infty$ due to the hypothesis of the lemma and

$$\begin{aligned} \big |f^{(1,0)}(x,e,\theta )^{\top }(\xi _{2}-\xi _{1})\big |^{2}\le & {} K_{\sigma }\big |f^{(1,0)}(x,e,\theta )^{\top }(y-x)\big |^{2} \\= & {} K_{\sigma }\big |f(y,e,\theta )-f(x,e,\theta )-{\mathcal {O}}(\Vert y-x\Vert ^{2})\big |^{2} \\\le & {} 3K_{\sigma }|f(y,e,\theta )|^{2}+3K_{\sigma }|f(x,e,\theta )|^{2}+{\mathcal {O}}(\Vert y-x\Vert ^{4}) \end{aligned}$$

where the positive constant $K_{\sigma }$ satisfies $\xi _{2}-\xi _{1}=\sqrt{K_{\sigma }}(y-x)$ for $y\in \big \{\tilde{x}\in {\mathcal {X}}:\Vert \tilde{x}-x\Vert \le \sigma \big \}$. In fact, for given $\xi _{1}$, $\xi _{2}$ and *x*, we can find a $\sigma$ to be such that the hypothesis of the lemma is satisfied, and then determine *y* and $K_{\sigma }$.

To estimate the term $\beta _{\epsilon }$, we divide [0, *s*] into some intervals depending of a given size $\vartriangle$ and define a function $\bar{P}^{\vartriangle }_{s}$ by

$$\begin{aligned} \bar{P}^{\vartriangle }_{s}=\bar{P}_{[\frac{s}{\vartriangle }]\vartriangle } \end{aligned}$$

where $\big [\frac{s}{\vartriangle }\big ]$ is the integer part of $\frac{s}{\vartriangle }$. Then, using the Cauchy–Schwartz inequality we have

$$\begin{aligned} \beta _{\epsilon }= & {} \sup _{0\le t_{1}\le T}2\bigg |\int _{0}^{t_{1}}\big (\bar{P}_{s}(1-\bar{P}_{s})h(Z_{s/\epsilon })-\bar{P}^{\vartriangle }_{s}(1-\bar{P}^{\vartriangle }_{s})h(Z_{s/\epsilon }) \\&\ \ \ \ \ \ \ \ \ \ \ \ \ \ \ \ \ \ +\bar{P}^{\vartriangle }_{s}(1-\bar{P}^{\vartriangle }_{s})h(Z_{s/\epsilon })-\bar{P}^{\vartriangle }_{s}(1-\bar{P}^{\vartriangle }_{s})\bar{h} \\&\ \ \ \ \ \ \ \ \ \ \ \ \ \ \ \ \ \ +\bar{P}^{\vartriangle }_{s}(1-\bar{P}^{\vartriangle }_{s})\bar{h}-\bar{P}_{s}(1-\bar{P}_{s})\bar{h}\big )ds\bigg |^{2} \\\le & {} \sup _{0\le t_{1}\le T}6\bigg |\int _{0}^{t_{1}}\big (\bar{P}_{s}(1-\bar{P}_{s})h(Z_{s/\epsilon })-\bar{P}^{\vartriangle }_{s}(1-\bar{P}^{\vartriangle }_{s})h(Z_{s/\epsilon })\big )ds\bigg |^{2} \\&+\sup _{0\le t_{1}\le T}6\bigg |\int _{0}^{t_{1}}\big (\bar{P}^{\vartriangle }_{s}(1-\bar{P}^{\vartriangle }_{s})h(Z_{s/\epsilon })-\bar{P}^{\vartriangle }_{s}(1-\bar{P}^{\vartriangle }_{s})\bar{h}\big )ds\bigg |^{2} \\&+\sup _{0\le t_{1}\le T}6\bigg |\int _{0}^{t_{1}}\big (\bar{P}^{\vartriangle }_{s}(1-\bar{P}^{\vartriangle }_{s})\bar{h}-\bar{P}_{s}(1-\bar{P}_{s})\bar{h}\big )ds\bigg |^{2} \\\le & {} \sup _{0\le t_{1}\le T}6\int _{0}^{t_{1}}\big |(\bar{P}_{s}-\bar{P}^{\vartriangle }_{s})(1-\bar{P}_{s}-\bar{P}^{\vartriangle }_{s})\big |^{2}ds\int _{0}^{T}\big |h(Z_{s/\epsilon })\big |^{2}ds \\&+\sup _{0\le t_{1}\le T}6\bigg |\int _{0}^{t_{1}}\bar{P}^{\vartriangle }_{s}(1-\bar{P}^{\vartriangle }_{s})\big (h(Z_{s/\epsilon })-\bar{h}\big )ds\bigg |^{2} \\&+\sup _{0\le t_{1}\le T}6\bar{h}^{2}T\int _{0}^{T}\big |(\bar{P}^{\vartriangle }_{s}-\bar{P}_{s})(1-\bar{P}^{\vartriangle }_{s}-\bar{P}_{s})\big |^{2}ds \\\le & {} \sup _{0\le t_{1}\le T}6\int _{0}^{t_{1}}|\bar{P}_{s}-\bar{P}^{\vartriangle }_{s}|^{2}ds\int _{0}^{T}\big |h(Z_{s/\epsilon })\big |^{2}ds \\&+\sup _{0\le t_{1}\le T}6\bigg |\int _{0}^{t_{1}}\bar{P}^{\vartriangle }_{s}(1-\bar{P}^{\vartriangle }_{s})\big (h(Z_{s/\epsilon })-\bar{h}\big )ds\bigg |^{2} \\&+\sup _{0\le t_{1}\le T}6\bar{h}^{2}T\int _{0}^{T}|\bar{P}^{\vartriangle }_{s}-\bar{P}_{s}|^{2}ds \\\le & {} (3\alpha _{\epsilon }T+6\bar{h}^{2}T^{2})\sup _{0\le s\le T}|\bar{P}_{s}-\bar{P}^{\vartriangle }_{s}|^{2} \\&+\sup _{0\le t_{1}\le T}6\bigg |\int _{0}^{t_{1}}\bar{P}^{\vartriangle }_{s}(1-\bar{P}^{\vartriangle }_{s})\big (h(Z_{s/\epsilon })-\bar{h}\big )ds\bigg |^{2}. \end{aligned}$$

Following the proof of Liu and Krstic (2012, (4.110) and (4.111) in pp. 73–74), we immediately have, for all $p\in [0,1]$ and for $\mu$-almost every $z\in {\mathcal {Z}}\setminus {\mathcal {Z}}_{0}$,

$$\begin{aligned} {\mathbb {P}}_{(p,z)}\bigg \{\lim _{\epsilon \rightarrow 0}\sup _{0\le t_{1}\le T}6\bigg |\int _{0}^{t_{1}}\bar{P}^{\vartriangle }_{s}(1-\bar{P}^{\vartriangle }_{s})\big (h(Z_{s/\epsilon })-\bar{h}\big )ds\bigg |^{2}=0\bigg \}=1. \end{aligned}$$

By this limit and

$$\begin{aligned} \lim _{\vartriangle \rightarrow 0}\sup _{0\le s\le T}|\bar{P}_{s}-\bar{P}^{\vartriangle }_{s}|^{2}=0, \end{aligned}$$

we obtain that for all $p\in [0,1]$ and for $\mu$-almost every $z\in {\mathcal {Z}}\setminus {\mathcal {Z}}_{0}$

c.3 $$\begin{aligned} {\mathbb {P}}_{(p,z)}\Big \{\lim _{\epsilon \rightarrow 0}\beta _{\epsilon }=0\Big \}=1. \end{aligned}$$

Hence, by (c.2) and (c.3), for all $p\in [0,1]$ and for $\mu$-almost every $z\in {\mathcal {Z}}\setminus {\mathcal {Z}}_{0}$

$$\begin{aligned} {\mathbb {P}}_{(p,z)}\bigg \{\lim _{\epsilon \rightarrow 0}\sup _{0\le t_{1}\le T}|P^{\epsilon }_{t_{1}}-\bar{P}_{t_{1}}|^{2}=0\bigg \}=1 \end{aligned}$$

which implies the desired result of the lemma.

The proof is complete. $\square$

Secondly, following the proof of Liu and Krstic (2012, the assertion (i) of Theorem 4.1, see pp. 65–66), the finite-time approximation (c.1) can be extended to arbitrarily long time intervals as the following lemma shows. For the sake of self-containedness, we give the proof.

### Lemma 5

Assume that the hypothesis of Lemma 4 is satisfied. Then for all $\delta \in (0,1)$, the solutions of (2.7) and (2.9) satisfy

c.4 $$\begin{aligned} {\mathbb {P}}_{(p,z)}\Big \{\lim _{\epsilon \rightarrow 0}\inf \big \{t_{1}\ge 0: |P^{\epsilon }_{t_{1}}-\bar{P}_{t_{1}}|>\delta \big \}=+\infty \Big \}=1 \end{aligned}$$

for all $p\in [0,1]$ and for $\mu$-almost every $z\in {\mathcal {Z}}\setminus {\mathcal {Z}}_{0}$.

### Proof

Define a set ${\widetilde{\varOmega }}=\big \{\omega : \lim _{\epsilon \rightarrow 0}\sup _{0\le t_{1}\le T}|P^{\epsilon }_{t_{1}}(\omega )-\bar{P}_{t_{1}}|=0,\ \forall \ T\in {\mathbb {N}}\big \}$. From Lemma 4, it follows that ${\mathbb {P}}\{{\widetilde{\varOmega }}\}=1$. Let stoping time $\tau _{\delta }^{\epsilon }=\inf \big \{t_{1}\ge 0: |P^{\epsilon }_{t_{1}}-\bar{P}_{t_{1}}|>\delta \big \}$ for $\delta \in (0,1)$ and $\epsilon >0$. Notice that $P^{\epsilon }_{0}-\bar{P}_{0}=0$. By the continuity of the trajectories, $\tau _{\delta }^{\epsilon }\in (0,+\infty ]$ for all $\delta$ and $\epsilon$. Typically, $|P^{\epsilon }_{\tau _{\delta }^{\epsilon }}-\bar{P}_{\tau _{\delta }^{\epsilon }}|=\delta$ provided that $\tau _{\delta }^{\epsilon }<+\infty$.

For fixed $\delta \in (0,1)$ and any $\omega \in {\widetilde{\varOmega }}$, we get that for any $T>0$, there exists ${\tilde{\epsilon }}(\omega ,\delta ,T)>0$ such that $\tau _{\delta }^{\epsilon }(\omega )>T$ for any $0<\epsilon <{\tilde{\epsilon }}(\omega ,\delta ,T)$. It further implies that $\lim _{\epsilon \rightarrow 0}\tau _{\delta }^{\epsilon }(\omega )=+\infty$. From the fact ${\mathbb {P}}({\widetilde{\varOmega }})=1$, it follows that ${\mathbb {P}}\big \{\lim _{\epsilon \rightarrow 0}\tau _{\delta }^{\epsilon }=+\infty \big \}=1$.

The proof is complete. $\square$

Finally, from the averaged system (2.9), we have

$$\begin{aligned} \lim _{t_{1}\rightarrow +\infty }\bar{P}_{t_{1}}=p^{*} \end{aligned}$$

with $p^{*}=0\ \text {or}\ 1$, which is determined by the sign of $\partial _{y}{\mathcal {S}}_{x}(y)|_{y=x}(\xi _{2}-\xi _{1})$. It means that for every $\delta \in (0,1)$, there exists a constant $T_{\delta }>0$ such that

$$\begin{aligned} \sup _{t_{1}\ge T_{\delta }}|\bar{P}_{t_{1}}-p^{*}|<\frac{\delta }{2}. \end{aligned}$$

From this, the event

$$\begin{aligned} \bigg \{\sup _{t_{1}\ge T_{\delta }}|P^{\epsilon }_{t_{1}}-p^{*}|>\delta \bigg \}= & {} \bigcup _{t_{1}\ge T_{\delta }}\big \{|P^{\epsilon }_{t_{1}}-\bar{P}_{t_{1}}+\bar{P}_{t_{1}}-p^{*}|>\delta \big \} \\\subseteq & {} \bigcup _{t_{1}\ge T_{\delta }}\bigg \{|P^{\epsilon }_{t_{1}}-\bar{P}_{t_{1}}|>\frac{\delta }{2}\bigg \} \\\subseteq & {} \bigcup _{t_{1}\ge 0}\bigg \{|P^{\epsilon }_{t_{1}}-\bar{P}_{t_{1}}|>\frac{\delta }{2}\bigg \} \\= & {} \bigg \{\sup _{t_{1}\ge 0}|P^{\epsilon }_{t_{1}}-\bar{P}_{t_{1}}|>\frac{\delta }{2}\bigg \}, \end{aligned}$$

and then the stopping time

$$\begin{aligned} \inf \big \{t_{1}\ge T_{\delta }: |P^{\epsilon }_{t_{1}}-p^{*}|>\delta \big \}\ge \inf \bigg \{t_{1}\ge 0: |P^{\epsilon }_{t_{1}}-\bar{P}_{t_{1}}|>\frac{\delta }{2}\bigg \}. \end{aligned}$$

Thus, it follows from (c.4) that

$$\begin{aligned} {\mathbb {P}}_{(p,z)}\Big \{\lim _{\epsilon \rightarrow 0}\inf \big \{t_{1}\ge T_{\delta }:|P^{\epsilon }_{t_{1}}-p^{*}|>\delta \big \}=+\infty \Big \}=1 \end{aligned}$$

which is equivalent to (2.10).

The proof of Lemma 1 is complete.

## Appendix D Derivation of (2.12)

On $t_{2}$-timescale, (2.7) becomes

d.1 $$\begin{aligned} {\dot{P}}^{\epsilon }_{t_{2}}= & {} \frac{1}{2}P^{\epsilon }_{t_{2}}(1-P^{\epsilon }_{t_{2}})\big (\xi _{2}^{\top }f^{(2,0)}(x,e_{t_{2}/\epsilon ^{2}},\theta _{t_{2}/\epsilon ^{2}})\xi _{2}-\xi _{1}^{\top }f^{(2,0)}(x,e_{t_{2}/\epsilon ^{2}},\theta _{t_{2}/\epsilon ^{2}})\xi _{1} \nonumber \\&\ \ \ \ \ \ \ \ \ \ \ \ \ \ \ \ \ \ \ \ +2e^{(1)\top }_{t_{2}/\epsilon ^{2}}f^{(1,1)}(x,e_{t_{2}/\epsilon ^{2}},\theta _{t_{2}/\epsilon ^{2}})(\xi _{2}-\xi _{1})\big )+{\mathcal {O}}(\epsilon ) \\:= & {} P^{\epsilon }_{t_{2}}(1-P^{\epsilon }_{t_{2}})\phi (x,e_{t_{2}/\epsilon ^{2}},\theta _{t_{2}/\epsilon ^{2}},e^{(1)}_{t_{2}/\epsilon ^{2}})+{\mathcal {O}}(\epsilon ). \nonumber \end{aligned}$$

The displacement of the trajectory $P^{\epsilon }_{t_{2}}$ starting from *p* over a small time $\vartriangle$ is

$$\begin{aligned} P^{\epsilon }_{\vartriangle }-p= & {} \int _{0}^{\vartriangle }\big (P^{\epsilon }_{s}(1-P^{\epsilon }_{s})\phi (x,e_{s/\epsilon ^{2}},\theta _{s/\epsilon ^{2}},e^{(1)}_{s/\epsilon ^{2}})+{\mathcal {O}}(\epsilon )\big )ds \\= & {} \int _{0}^{\vartriangle }\big (p(1-p)\phi (x,e_{s/\epsilon ^{2}},\theta _{s/\epsilon ^{2}},e^{(1)}_{s/\epsilon ^{2}})+{\mathcal {O}}(\epsilon )\big )ds \\&+\int _{0}^{\vartriangle }\big (P^{\epsilon }_{s}(1-P^{\epsilon }_{s})-p(1-p)\big )\phi (x,e_{s/\epsilon ^{2}},\theta _{s/\epsilon ^{2}},e^{(1)}_{s/\epsilon ^{2}})ds \\= & {} \vartriangle \bigg (\frac{\epsilon ^{2}}{\vartriangle }\int _{0}^{\frac{\vartriangle }{\epsilon ^{2}}}p(1-p)\phi (x,e_{s},\theta _{s},e^{(1)}_{s})ds\bigg )+{\mathcal {O}}(\epsilon ,\vartriangle ), \end{aligned}$$

where the high order term ${\mathcal {O}}(\epsilon ,\vartriangle )$ converges to zero as $\vartriangle \rightarrow 0$ and $\epsilon \rightarrow 0$. Following from a standard argument as Freidlin and Wentzell (2012, Chapter 7), we claim that the solutions of $P^{\epsilon }_{t_{2}}$ converges to that of $\bar{P}_{t_{2}}$ of the following averaged system

d.2 $$\begin{aligned} \dot{\bar{P}}_{t_{2}}=\bar{P}_{t_{2}}(1-\bar{P}_{t_{2}})\langle \phi (x,e_{s},\theta _{s},e^{(1)}_{s})\rangle _{+\infty }, \ \ \bar{P}_{0}=p, \end{aligned}$$

on every finite time interval as $\epsilon \rightarrow 0$, where $\langle \cdot \rangle _{+\infty }$ means the time average for $t_{2}/\epsilon ^{2}\rightarrow +\infty$ as $\epsilon \rightarrow 0$. This result further can be extended to arbitrarily long time intervals in the same manner as in Lemmas 4 and 5.

To arrive the averaged system (2.12), we need the time evolution of $e^{(1)}_{t}$ in fast time *t*. From now on, we will use the simplified symbols $(N_{t},e_{t})$ to replace $(N^{0}_{t},e^{0}_{t})$ used in Sect. 2.5.1. Consider the series expansion of model (2.5) with $P_{t}=p$ for all *t*, the first-order terms in $\epsilon$ of (2.5a), (2.5c) and (2.5d) are

$$\begin{aligned} {\dot{N}}^{(1)}_{t}= & {} fN^{(1)}_{t}+\big (e^{(1)\top }_{t}f^{(0,1)}+\big ((1-p)\xi _{1}+p\xi _{2}\big )^{\top }f^{(1,0)}\big )N_{t}, \\ {\dot{e}}^{(1)}_{1,t}= & {} e^{(1)\top }_{t}G^{(1)}_{1}+H_{1}N^{(1)}_{t}+\big (e^{(1)\top }_{t}H^{(0,1)}_{1}+\big ((1-p)\xi _{1}+p\xi _{2}\big )^{\top }H^{(1,0)}_{1}\big )N_{t}, \\ e^{(1)}_{2,t}= & {} e^{(1)\top }_{t}G^{(1)}_{2}+H_{2}N^{(1)}_{t}+\big (e^{(1)\top }_{t}H^{(0,1)}_{2}+\big ((1-p)\xi _{1}+p\xi _{2}\big )^{\top }H^{(1,0)}_{2}\big )N_{t}, \\ {\dot{\theta }}_{t}= & {} A(\theta _{t})+B(\theta _{t}){\dot{W}}_{t}, \end{aligned}$$

where functions *f*, $f^{(0,1)}$, $f^{(1,0)}$, $G^{(1)}_{i}$, $H_{i}$, $H^{(0,1)}_{i}$ and $H^{(1,0)}_{i}$ are all evaluated at $(x,e_{t},\theta _{t})$, and where $(N_{t},e_{t},\theta _{t})$ are calculated from (2.6) that is independent of *p*. Generally, it is impossible to obtain an explicit expression for $e^{(1)}_{t}$ by solving the above equations. Instead, we focus on an equivalent relation which will be employed to derive the averaged system (2.12).

Consider the second-order terms in $\epsilon$ of the series expansions of ${\mathcal {S}}_{x_{1}}(x_{2})$ and ${\mathcal {S}}_{x_{1}}(x_{1})$, we have

d.3 $$\begin{aligned} \begin{aligned} \lim _{\epsilon \rightarrow 0}\frac{{\mathcal {S}}_{x_{1}}(x_{2})}{\epsilon ^{2}}&=\lim _{\epsilon \rightarrow 0}\frac{{\mathcal {S}}_{x+\epsilon \xi _{1}}(x+\epsilon \xi _{2})-{\mathcal {S}}_{x+\epsilon \xi _{1}}(x+\epsilon \xi _{1})}{\epsilon ^{2}} \\&=\frac{1}{2}\big \langle \xi _{2}^{\top }f^{(2,0)}\xi _{2}-\xi _{1}^{\top }f^{(2,0)}\xi _{1}+2\hat{e}^{(1)\top }_{t}f^{(1,1)}(\xi _{2}-\xi _{1})\big \rangle _{+\infty } \end{aligned} \end{aligned}$$

where $\hat{e}^{(1)}_{t}$ is calculated from

d.4 $$\begin{aligned} \begin{aligned} \dot{\hat{N}}^{(1)}_{t}&=f\hat{N}^{(1)}_{t}+\big (\hat{e}^{(1)\top }_{t}f^{(0,1)}+\xi _{2}^{\top }f^{(1,0)}\big )N_{t}, \\ \dot{\hat{e}}^{(1)}_{1,t}&=\hat{e}^{(1)\top }_{t}G^{(1)}_{1}+H_{1}\hat{N}^{(1)}_{t}+\big (\hat{e}^{(1)\top }_{t}H^{(0,1)}_{1}+\xi _{2}^{\top }H^{(1,0)}_{1}\big )N_{t}, \\ \hat{e}^{(1)}_{2,t}&=\hat{e}^{(1)\top }_{t}G^{(1)}_{2}+H_{2}\hat{N}^{(1)}_{t}+\big (\hat{e}^{(1)\top }_{t}H^{(0,1)}_{2}+\xi _{2}^{\top }H^{(1,0)}_{2}\big )N_{t}, \\ {\dot{\theta }}_{t}&= A(\theta _{t})+B(\theta _{t}){\dot{W}}_{t}. \end{aligned} \end{aligned}$$

Similarly, consider the second-order terms in $\epsilon$ of the series expansions of ${\mathcal {S}}_{x_{2}}(x_{1})$ and ${\mathcal {S}}_{x_{2}}(x_{2})$, we have

d.5 $$\begin{aligned} \begin{aligned} \lim _{\epsilon \rightarrow 0}\frac{{\mathcal {S}}_{x_{2}}(x_{1})}{\epsilon ^{2}}&=\lim _{\epsilon \rightarrow 0}\frac{{\mathcal {S}}_{x+\epsilon \xi _{2}}(x+\epsilon \xi _{1})-{\mathcal {S}}_{x+\epsilon \xi _{2}}(x+\epsilon \xi _{2})}{\epsilon ^{2}} \\&=-\,\frac{1}{2}\big \langle \xi _{2}^{\top }f^{(2,0)}\xi _{2}-\xi _{1}^{\top }f^{(2,0)}\xi _{1}+2{\check{e}}^{(1)\top }_{t}f^{(1,1)}(\xi _{2}-\xi _{1})\big \rangle _{+\infty } \end{aligned} \end{aligned}$$

where ${\check{e}}^{(1)}_{t}$ is calculated from

d.6 $$\begin{aligned} \begin{aligned} \dot{{\check{N}}}^{(1)}_{t}&=f{\check{N}}^{(1)}_{t}+\big ({\check{e}}^{(1)\top }_{t}f^{(0,1)}+\xi _{1}^{\top }f^{(1,0)}\big )N_{t}, \\ \dot{{\check{e}}}^{(1)}_{1,t}&={\check{e}}^{(1)\top }_{t}G^{(1)}_{1}+H_{1}{\check{N}}^{(1)}_{t}+\big ({\check{e}}^{(1)\top }_{t}H^{(0,1)}_{1}+\xi _{1}^{\top }H^{(1,0)}_{1}\big )N_{t}, \\ {\check{e}}^{(1)}_{2,t}&={\check{e}}^{(1)\top }_{t}G^{(1)}_{2}+H_{2}{\check{N}}^{(1)}_{t}+\big ({\check{e}}^{(1)\top }_{t}H^{(0,1)}_{2}+\xi _{1}^{\top }H^{(1,0)}_{2}\big )N_{t}, \\ {\dot{\theta }}_{t}&= A(\theta _{t})+B(\theta _{t}){\dot{W}}_{t}. \end{aligned} \end{aligned}$$

Denote

$$\begin{aligned} \widetilde{N}^{(1)}_{t}=(1-p)\hat{N}^{(1)}_{t}+p{\check{N}}^{(1)}_{t},\ \tilde{e}^{(1)}_{t}=(1-p)\hat{e}^{(1)}_{t}+p{\check{e}}^{(1)}_{t}. \end{aligned}$$

By (d.3) and (d.5), we get

d.7 $$\begin{aligned} \begin{aligned}&\lim _{\epsilon \rightarrow 0}\frac{(1-p){\mathcal {S}}_{x_{1}}(x_{2})-p{\mathcal {S}}_{x_{2}}(x_{1})}{\epsilon ^{2}} \\&=\frac{1}{2}\big \langle \xi _{2}^{\top }f^{(2,0)}\xi _{2}-\xi _{1}^{\top }f^{(2,0)}\xi _{1}+2\tilde{e}^{(1)\top }_{t}f^{(1,1)}(\xi _{2}-\xi _{1})\big \rangle _{+\infty }. \end{aligned} \end{aligned}$$

It follows from (d.4) and (d.6) that the dynamics of $\widetilde{N}^{(1)}_{t}$ and $\tilde{e}^{(1)}_{t}$ are given by

$$\begin{aligned} \dot{\widetilde{N}^{(1)}_{t}}= & {} f\widetilde{N}^{(1)}_{t}+\Big (\tilde{e}^{(1)\top }_{t}f^{(0,1)}+\big ((1-p)\xi _{1}+p\xi _{2}\big )^{\top }f^{(1,0)}\Big )N_{t},\\ \dot{\tilde{e}}^{(1)}_{1,t}= & {} \tilde{e}^{(1)\top }_{t}G^{(1)}_{1}+H_{1}\widetilde{N}^{(1)}_{t}+\Big (\tilde{e}^{(1)\top }_{t}H^{(0,1)}_{1}+\big ((1-p)\xi _{1}+p\xi _{2}\big )^{\top }H^{(1,0)}_{1}\Big )N_{t}, \\ \tilde{e}^{(1)}_{2,t}= & {} \tilde{e}^{(1)\top }_{t}G^{(1)}_{2}+H_{2}\widetilde{N}^{(1)}_{t}+\Big (\tilde{e}^{(1)\top }_{t}H^{(0,1)}_{2}+\big ((1-p)\xi _{1}+p\xi _{2}\big )^{\top }H^{(1,0)}_{2}\Big )N_{t}, \\ {\dot{\theta }}_{t}= & {} A(\theta _{t})+B(\theta _{t}){\dot{W}}_{t}, \end{aligned}$$

which shows that $(\widetilde{N}^{(1)}_{t},\tilde{e}^{(1)}_{t})$ has the same dynamics as $(N^{(1)}_{t},e^{(1)}_{t})$.

Since by (d.1), (d.2) and (d.7) we obtain that

d.8 $$\begin{aligned} \frac{\dot{\bar{P}}_{t_{2}}}{\bar{P}_{t_{2}}(1-\bar{P}_{t_{2}})}=\lim _{\epsilon \rightarrow 0}\frac{(1-\bar{P}_{t_{2}}){\mathcal {S}}_{x_{1}}(x_{2})-\bar{P}_{t_{2}}{\mathcal {S}}_{x_{2}}(x_{1})}{\epsilon ^{2}}, \end{aligned}$$

indicating that now we can express the dynamics of $\bar{P}_{t_{2}}$ in terms of the expansions of the two invasion fitnesses. From ${\mathcal {S}}_{x}(x)=0$ applied to the second-order terms in its series expansion we have

d.9 $$\begin{aligned} {\mathcal {C}}_{11}+{\mathcal {C}}_{22}+{\mathcal {C}}_{12}+{\mathcal {C}}_{21}=0, \end{aligned}$$

where $\{{\mathcal {C}}_{ij}\}_{i,j\in \{1,2\}}$ are the second derivatives of invasion fitness (ref to (2.13)). Using the second-order terms in $\epsilon$ of the series expansions of ${\mathcal {S}}_{x_{1}}(x_{2})$ and ${\mathcal {S}}_{x_{2}}(x_{1})$ and perusing (d.9), (d.8) is equivalent to

$$\begin{aligned} \frac{\dot{\bar{P}}_{t_{2}}}{\bar{P}_{t_{2}}(1-\bar{P}_{t_{2}})}= & {} (1-\bar{P}_{t_{2}})(\xi ^{\top }_{1}{\mathcal {C}}_{11}\xi _{1}+\xi ^{\top }_{2}{\mathcal {C}}_{22}\xi _{2}+2\xi ^{\top }_{1}{\mathcal {C}}_{21}\xi _{2}) \\&-\,\bar{P}_{t_{2}}(\xi ^{\top }_{2}{\mathcal {C}}_{11}\xi _{2}+\xi ^{\top }_{1}{\mathcal {C}}_{22}\xi _{1}+2\xi ^{\top }_{2}{\mathcal {C}}_{21}\xi _{1}) \\= & {} (1-\bar{P}_{t_{2}})\big (\xi ^{\top }_{2}{\mathcal {C}}_{22}\xi _{2}-\xi ^{\top }_{1}{\mathcal {C}}_{22}\xi _{1}+2\xi ^{\top }_{1}{\mathcal {C}}_{21}(\xi _{2}-\xi _{1})\big ) \\&-\,\bar{P}_{t_{2}}\big (-\xi ^{\top }_{2}{\mathcal {C}}_{22}\xi _{2}+\xi ^{\top }_{1}{\mathcal {C}}_{22}\xi _{1}-2\xi ^{\top }_{2}{\mathcal {C}}_{21}(\xi _{2}-\xi _{1})\big ) \\= & {} \xi _{2}^{\top }{\mathcal {C}}_{22}\xi _{2}-\xi _{1}^{\top }{\mathcal {C}}_{22}\xi _{1}+2\big ((1-\bar{P}_{t_{2}})\xi _{1}+\bar{P}_{t_{2}}\xi _{2}\big )^{\top }{\mathcal {C}}_{21}(\xi _{2}-\xi _{1}). \end{aligned}$$

To complete the derivation of (2.12), the right side of the last equality needs to be simplified via the following three steps: firstly,

$$\begin{aligned} \xi _{2}^{\top }{\mathcal {C}}_{22}\xi _{2}-\xi _{1}^{\top }{\mathcal {C}}_{22}\xi _{1}=(\xi _{2}+\xi _{1})^{\top }{\mathcal {C}}_{22}(\xi _{2}-\xi _{1}); \end{aligned}$$

secondly,

$$\begin{aligned}&2\big ((1-\bar{P}_{t_{2}})\xi _{1}+\bar{P}_{t_{2}}\xi _{2}\big )^{\top }{\mathcal {C}}_{21}(\xi _{2}-\xi _{1}) \\&=\big ((1-\bar{P}_{t_{2}})\xi _{1}+\bar{P}_{t_{2}}\xi _{2}\big )^{\top }{\mathcal {C}}_{21}(\xi _{2}-\xi _{1})+(\xi _{2}-\xi _{1})^{\top }{\mathcal {C}}_{12}\big ((1-\bar{P}_{t_{2}})\xi _{1}+\bar{P}_{t_{2}}\xi _{2}\big ) \\&=\xi _{1}^{\top }{\mathcal {C}}_{21}(\xi _{2}-\xi _{1})+(\xi _{2}-\xi _{1})^{\top }{\mathcal {C}}_{12}\xi _{1}-\bar{P}_{t_{2}}(\xi _{2}-\xi _{1})^{\top }({\mathcal {C}}_{22}+{\mathcal {C}}_{11})(\xi _{2}-\xi _{1}); \end{aligned}$$

finally,

$$\begin{aligned}&\xi _{1}^{\top }{\mathcal {C}}_{21}(\xi _{2}-\xi _{1})+(\xi _{2}-\xi _{1})^{\top }{\mathcal {C}}_{12}\xi _{1} \\&=(\xi _{2}+\xi _{1})^{\top }{\mathcal {C}}_{21}(\xi _{2}-\xi _{1})-(\xi _{2}-\xi _{1})^{\top }{\mathcal {C}}_{21}(\xi _{2}-\xi _{1}) \\&=(\xi _{2}+\xi _{1})^{\top }{\mathcal {C}}_{21}(\xi _{2}-\xi _{1})+\frac{1}{2}(\xi _{2}-\xi _{1})^{\top }({\mathcal {C}}_{22}+{\mathcal {C}}_{11})(\xi _{2}-\xi _{1}). \end{aligned}$$

Therefore,

$$\begin{aligned} \frac{\dot{\bar{P}}_{t_{2}}}{\bar{P}_{t_{2}}(1-\bar{P}_{t_{2}})}= & {} (\xi _{2}+\xi _{1})^{\top }({\mathcal {C}}_{22}+{\mathcal {C}}_{21})(\xi _{2}-\xi _{1}) \\&+\,\Big (\frac{1}{2}-\bar{P}_{t_{2}}\Big )(\xi _{2}-\xi _{1})^{\top }({\mathcal {C}}_{22}+{\mathcal {C}}_{11})(\xi _{2}-\xi _{1}) \end{aligned}$$

which is the desired result.

## Appendix E Proof of Lemma 2

The first assertion can be proven in the same manner as in Lemma 1, in which the associated averaged system becomes (2.12). The details are left to the reader.

Next, we focus on the proof of the second assertion. Let $P^{\epsilon }_{0}=\bar{P}_{0}=p$ and $Z_{0}=z$. Denote

$$\begin{aligned} p^{*}_{0}=0,\ p^{*}_{1/2}=\frac{{\mathcal {S}}_{x_{1}}(x_{2})}{{\mathcal {S}}_{x_{1}}(x_{2})+{\mathcal {S}}_{x_{2}}(x_{1})},\ p^{*}_{1}=1. \end{aligned}$$

One hand, from the averaged system (2.12), the bistability indicates that $p^{*}_{1/2}\in (0,1)$ is a saddle node such that

$$\begin{aligned} \lim _{t_{2}\rightarrow +\infty }\bar{P}_{t_{2}}=p^{*}_{0}\ \ \text {for all}\ p\in [0,p^{*}_{1/2}), \end{aligned}$$

and

$$\begin{aligned} \lim _{t_{2}\rightarrow +\infty }\bar{P}_{t_{2}}=p^{*}_{1}\ \ \text {for all}\ p\in (p^{*}_{1/2},1]. \end{aligned}$$

On the other hand, on $t_{2}$-timescale, we also have the similar result as Lemma 5, i.e., for all $\delta \in (0,1)$

e.1 $$\begin{aligned} {\mathbb {P}}_{(p,z)}\Big \{\lim _{\epsilon \rightarrow 0}\inf \big \{t_{2}\ge 0: |P^{\epsilon }_{t_{2}}-\bar{P}_{t_{2}}|>\delta \big \}=+\infty \Big \}=1 \end{aligned}$$

for all $p\in [0,1]\setminus \{p^{*}_{1/2}\}$ and for $\mu$-almost every $z\in {\mathcal {Z}}\setminus {\mathcal {Z}}_{0}$.

Let stopping times

$$\begin{aligned} \tau ^{\epsilon }_{0,\delta }:= & {} \inf \big \{t_{2}\ge T_{\delta }: |P^{\epsilon }_{t_{2}}-p^{*}_{0}|>\delta \big \},\\ \tau ^{\epsilon }_{1,\delta }:= & {} \inf \big \{t_{2}\ge T_{\delta }: |P^{\epsilon }_{t_{2}}-p^{*}_{1}|>\delta \big \}. \end{aligned}$$

For any $\varepsilon >0$, by (e.1), the continuity of trajectories and the Markov property, there is ${\tilde{\delta }}\in \big (0, \min \{p^{*}_{1/2},1-p^{*}_{1/2}\}\big )$ such that for every $\delta \in (0,{\tilde{\delta }})$, there exists $T_{\delta }>0$ such that

$$\begin{aligned} \tilde{q}^{0}_{(p,z)}:={\mathbb {P}}_{(p,z)}\Big \{\lim _{\epsilon \rightarrow 0}\tau ^{\epsilon }_{0,\delta }=+\infty \Big \}\ge 1-\varepsilon \ \ \text {for all}\ p\in [0,\delta ) \end{aligned}$$

and

$$\begin{aligned} \tilde{q}^{1}_{(p,z)}:={\mathbb {P}}_{(p,z)}\Big \{\lim _{\epsilon \rightarrow 0}\tau ^{\epsilon }_{1,\delta }=+\infty \Big \}\ge 1-\varepsilon \ \ \text {for all}\ p\in (1-\delta ,1]. \end{aligned}$$

We now show that $\tilde{q}^{0}_{(p,z)}+\tilde{q}^{1}_{(p,z)}=1$ for all $p\in [0,1]\setminus \{p^{*}_{1/2}\}$ and for $\mu$-almost every ${\mathcal {Z}}\setminus {\mathcal {Z}}_{0}$. For this we only need to prove that the interval $[\delta ,1-\delta ]$ is transient, i.e., almost every trajectory of $P^{\epsilon }_{t_{2}}$ will escape from $[\delta ,1-\delta ]$ after some finite times later. In fact, by (e.1), there exist $\widetilde{T}_{\delta }>0$ and ${\tilde{\epsilon }}>0$ such that ${\mathbb {P}}_{(p,z)}\big \{|P^{\epsilon }_{t_{2}}-\bar{P}_{t_{2}}|\le \frac{\delta }{2}\ \text {for all}\ 0<\epsilon \le {\tilde{\epsilon }}\ \text {and}\ t_{2}\ge \widetilde{T}_{\delta }\big \}\ge 1-\varepsilon$. Hence, for any $t_{2}\ge \widetilde{T}_{\delta }$ and $p\in (\delta ,1-\delta )$, the event

$$\begin{aligned} \big \{P^{\epsilon }_{t_{2}}\not \in [\delta ,1-\delta ]\big \}= & {} \big \{P^{\epsilon }_{t_{2}}-\bar{P}_{t_{2}}+\bar{P}_{t_{2}}\not \in [\delta ,1-\delta ]\big \} \\= & {} \big \{\bar{P}_{t_{2}}\not \in \big [\delta -(P^{\epsilon }_{t_{2}}-\bar{P}_{t_{2}}),1-\delta -(P^{\epsilon }_{t_{2}}-\bar{P}_{t_{2}})\big ]\big \} \\\supseteq & {} \bigg \{\bar{P}_{t_{2}}\not \in \bigg [\frac{\delta }{2},1-\frac{\delta }{2}\bigg ]\bigg \}, \end{aligned}$$

and then the stopping time

$$\begin{aligned} \inf \big \{t_{2}\ge 0: P^{\epsilon }_{t_{2}}\not \in [\delta ,1-\delta ]\big \}\le & {} \inf \big \{t_{2}\ge \widetilde{T}_{\delta }: P^{\epsilon }_{t_{2}}\not \in [\delta ,1-\delta ]\big \} \\\le & {} \inf \bigg \{t_{2}\ge \widetilde{T}_{\delta }: \bar{P}_{t_{2}}\not \in \bigg [\frac{\delta }{2},1-\frac{\delta }{2}\bigg ]\bigg \}, \end{aligned}$$

which implies that

$$\begin{aligned} {\mathbb {P}}_{(p,z)}\big \{\lim _{\epsilon \rightarrow 0}\inf \big \{t_{2}\ge 0: P^{\epsilon }_{t_{2}}\not \in [\delta ,1-\delta ]\big \}<+\infty \big \}\ge 1-\varepsilon . \end{aligned}$$

It follows from the Markov property that

$$\begin{aligned} {\mathbb {P}}_{(p,z)}\Big \{\lim _{\epsilon \rightarrow 0}\tau ^{\epsilon }_{0,\delta }=+\infty \ \ \text {or}\ \lim _{\epsilon \rightarrow 0}\tau ^{\epsilon }_{1,\delta }=+\infty \Big \}\ge 1-\varepsilon \end{aligned}$$

for all $p\in [0,1]\setminus \{p^{*}_{1/2}\}$ and for $\mu$-almost every ${\mathcal {Z}}\setminus {\mathcal {Z}}_{0}$. Since the $\varepsilon$ is taken arbitrarily, we claim that $\tilde{q}^{0}_{(p,z)}+\tilde{q}^{1}_{(p,z)}=1$.

For the case of $p=p^{*}_{1/2}$, we have $P^{\epsilon }_{t_{2}}\ne p^{*}_{1/2}$ some time later due to the continuity of solutions. Thus, the above analysis is still valid so that $\tilde{q}^{0}_{(p,z)}+\tilde{q}^{1}_{(p,z)}=1$ for all $p\in [0,1]$.

By the definitions of $\tau ^{\epsilon }_{0,\delta }$ and $\tau ^{\epsilon }_{1,\delta }$, $\tilde{q}^{0}_{(p,z)}+\tilde{q}^{1}_{(p,z)}=1$ is equivalent to (2.16). This proves the second assertion of Lemma 2.
